# Synergistic interactions between PLK1 and HDAC inhibitors in non-Hodgkin's lymphoma cells occur *in vitro* and *in vivo* and proceed through multiple mechanisms

**DOI:** 10.18632/oncotarget.15649

**Published:** 2017-02-23

**Authors:** Tri Nguyen, Rebecca Parker, Elisa Hawkins, Beata Holkova, Victor Yazbeck, Akhil Kolluri, Maciej Kmieciak, Mohamed Rahmani, Steven Grant

**Affiliations:** ^1^ Division of Hematology/Oncology, Department of Internal Medicine, Virginia Commonwealth University and the Massey Cancer Center, Richmond, VA, USA; ^2^ Departments of Biochemistry, Virginia Commonwealth University, Richmond, VA, USA; ^3^ Departments of Pharmacology, Virginia Commonwealth University, Richmond, VA, USA; ^4^ Virginia Institute for Molecular Medicine, Virginia Commonwealth University, Richmond, VA, USA; ^5^ Massey Cancer Center, Virginia Commonwealth University Health Sciences Center, Richmond, VA, USA

**Keywords:** non-Hodgkin's lymphoma, volasertib, belinostat

## Abstract

Interactions between the polo-like kinase 1 (PLK1) inhibitor volasertib and the histone deacetylase inhibitor (HDACI) belinostat were examined in diffuse large B-cell lymphoma (DLBCL) and mantle cell lymphoma (MCL) cells *in vitro* and *in vivo*. Exposure of DLBCL cells to very low concentrations of volasertib in combination with belinostat synergistically increased cell death (apoptosis). Similar interactions occurred in GC-, ABC-, double-hit DLBCL cells, MCL cells, bortezomib-resistant cells and primary lymphoma cells. Co-exposure to volasertib/belinostat induced a marked increase in M-phase arrest, phospho-histone H3, mitotic errors, cell death in M-phase, and DNA damage. Belinostat diminished c-Myc mRNA and protein expression, an effect significantly enhanced by volasertib co-exposure. c-Myc knock-down increased DNA damage and cell death in response to volasertib, arguing that c-Myc down-regulation plays a functional role in the lethality of this regimen. Notably, PLK1 knock-down in DLBCL cells significantly increased belinostat-induced M-phase accumulation, phospho-histone H3, γH2AX, and cell death. Co-administration of volasertib and belinostat dramatically reduced tumor growth in an ABC-DLBCL flank model (U2932) and a systemic double-hit lymphoma model (OCI-Ly18), accompanied by a pronounced increase in survival without significant weight loss or other toxicities. Together, these findings indicate that PLK1/HDAC inhibition warrants attention as a therapeutic strategy in NHL.

## INTRODUCTION

Non-Hodgkin's lymphoma (NHL) is a lympho-proliferative neoplasm that afflicts approximately 70,000 patients/year in the US, and is responsible for 20,000 deaths [1]. Certain aggressive forms of this disease, such as diffuse large B-cell lymphoma (DLBCL), particularly the ABC sub-type, and mantle cell lymphoma, contribute disproportionally to the death rate. In addition, molecular profiling has identified genetic NHL signatures which predict poor prognoses e.g., double-hit lymphomas displaying aberrant expression of Bcl-2, c-Myc, and/or Bcl-6 [2]. Despite recent advances such as the introduction of effective new targeted therapies for this disease (e.g., Ibrutinib) [3], patients with relapsed/refractory disease have a dismal prognosis [4]. Consequently, newer and more effective treatment strategies are urgently needed for these disorders.

Members of the serine-threonine polo-like kinase (PLK) family (PLK1-5) play key roles in multiple important cellular functions, including cell cycle progression, mitosis, cytokinesis, centriole duplication, and the DNA damage response (DDR) [5, 6]. PLK1 phosphorylates multiple substrates (e.g., Bora/Aurora A, cyclin B, cdc25C, Wee1) necessary for mitotic entry/progression [6, 7]. In response to DNA damage, PLK1 is required for mitotic entry following G_2_ arrest [5, 6]. It also contributes significantly to DNA repair (e.g., homologous recombination; HR) through activation of RAD51 [8]. Additionally, PLK1 regulates the mitotic spindle checkpoint [9]. Disruption of PLK1 function triggers multiple mitotic abnormalities, including aberrant mono-polar spindles, anaphase bridging, and arrest of cells in pro-metaphase (“Polo arrest”) accompanied by up-regulation of phospho-histone H3 and apoptosis [10, 11]. PLK1 inhibitors also induce “mitotic slippage”, defined as chromosome de-condensation and formation of nuclear envelopes without chromosome segregation and cytokinesis [12]. The observation that PLK1 is overexpressed in tumor cells [13–17], including non-Hodgkin's lymphoma [18], where it is associated with a poor prognosis [16, 17], but not in normal cells [19], makes it a logical target for therapeutic intervention. Notably, a PLK1 inhibitor, MLN0905, demonstrated significant pre-clinical activity in DLBCL models [20], and one of first PLK1 inhibitors to undergo clinical evaluation in humans, BI 2536 (Boerhinger-Ingelheim), demonstrated significant single-agent activity in patients with lymphoma (CTCL) in a phase I trial [21]. The second-generation PLK1 inhibitor BI 6727 (volasertib; BI) has superior pharmacokinetic characteristics compared to BI 2536 [22], and has recently been granted Orphan Drug designation [23]. Currently, experience with volasertib in DLBCL or other aggressive forms of NHL in general is lacking.

Histone deacetylase inhibitors (HDACIs) are epigenetic agents that acetylate histone tails, leading to a more open chromatin structure and modulation of the expression of genes implicated in cell survival and differentiation [24]. HDACIs kill transform cells through diverse mechanisms, including up-regulation of pro-apoptotic proteins (e.g., Bim) [25], death receptors induction [26], cell cycle checkpoint disruption and ROS generation among others [27, 28]. Like PLK1 inhibitors, HDACIs induce multiple mitotic abnormalities, including mitotic slippage [29]. Recently, attention has focused on selective induction of DNA damage in tumor cells stemming from diminished up-regulation of antioxidant proteins and/or disruption of DNA repair (e.g., both homologous recombination or non-homologous end-joining) [27, 30]. Notably, aberrant expression of histone modifying enzymes has been described in diffuse large-B cell lymphoma (DLBCL) [31]. In clinical practice, the HDACIs vorinostat and romidepsin have been approved for use in CTCL [32], and both romidepsin and the pan-HDACI belinostat received approval for the treatment of PTCL [33]. Most recently, the pan-HDACI mocetinostat obtained orphan drug status in DLBCL associated with p300 mutations [33].

Based upon the preceding considerations, PLK1 and HDAC inhibitors exhibit complementary mechanisms of action. For example, disruption of the mitotic spindle DNA damage checkpoint (e.g., by PLK1 inhibitors) [34] may potentiate the lethal consequences of HDACI-mediated DNA damage [27, 35]. Furthermore, both PLK1 [8] and HDACIs [27, 35] play key roles in regulating DNA repair proteins e.g., RAD51. In addition, both PLK1 inhibitors [12] and HDACIs [29] induce mitotic slippage, reflected by micronucleation and the presence of cells exhibiting ≥ 4N DNA. Finally, selective DNA damage induction by HDACIs [36] and high expression of PLK1 in tumor cells [13, 37] suggests that combinations might preferentially target neoplastic cells. Such factors, along with preliminary indications of PLK1 inhibitor activity in lymphoid malignancies [20, 21], raised the possibility that a PLK1/HDAC inhibitory strategy could be effective against DLBCL and related NHL cell types. To evaluate this possibility, we examined volasertib/belinostat interactions in various NHL lines, including several adverse risk sub-types e.g., ABC- and double-hit DLBCL cells. Our results indicate highly synergistic volasertib/belinostat interactions in NHL (but not normal) cells, events associated with striking M-phase arrest and mitotic abnormalities, c-Myc down-regulation, marked DNA damage, and cell death. Collectively, these findings argue that the PLK1/HDAC inhibitory strategy warrants further attention in NHL.

## RESULTS

### Volasertib and belinostat interact synergistically in multiple NHL cell types

**Figure 1:**
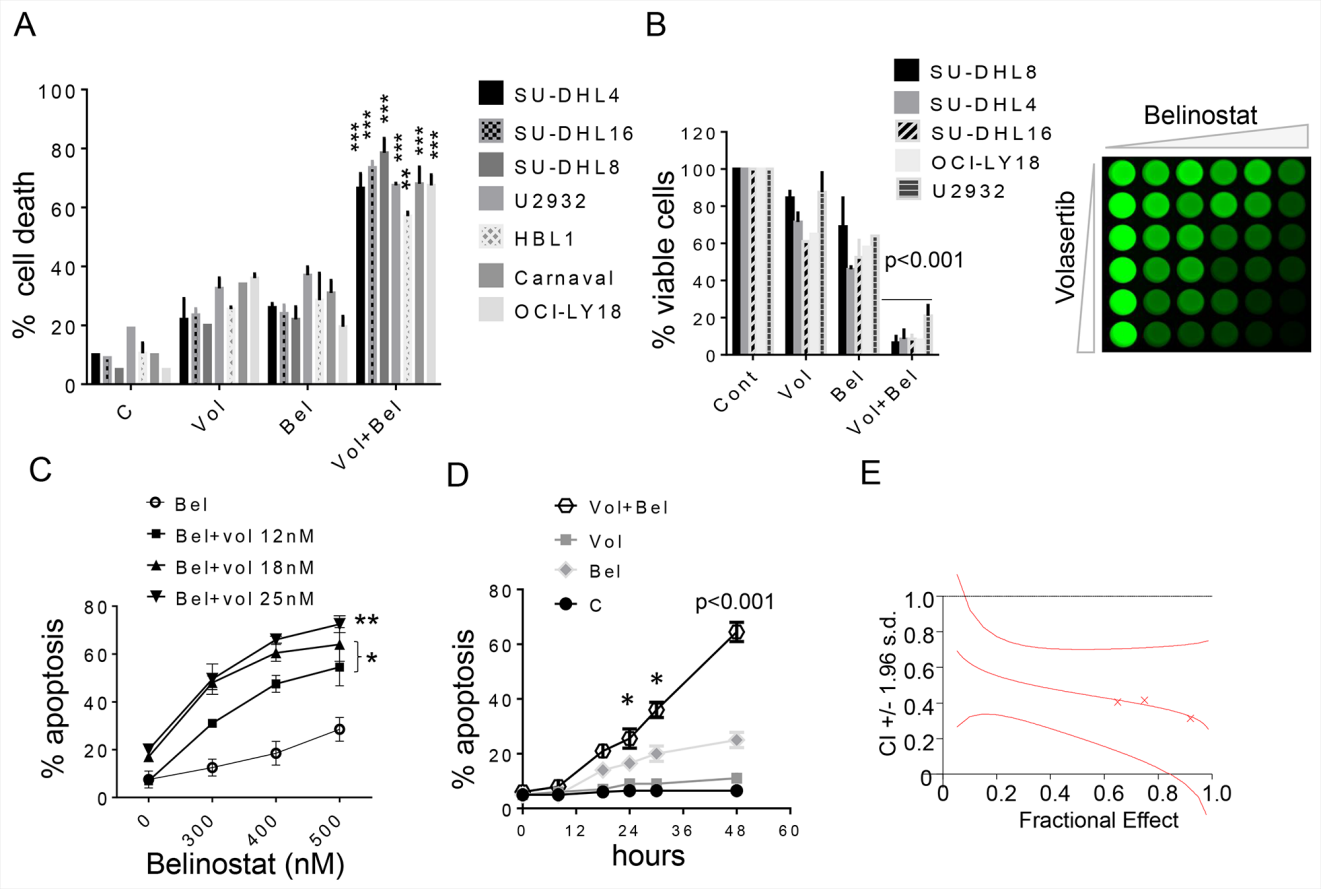
Belinostat dramatically increases volasertib lethality and inhibits cell growth in DLBCL cells **A**. Various NHL cell lines SU-DHL16, SU-DHL4, SU-DHL8 (GC subtype), HBL-1, U2932 (ABC-subtype), OCI-LY18, Carnaval (double hit) were exposed to volasertib (5-30 nmol/L) and belinostat (100-400 nmol/L) alone or together for 48 hr, after which cell death was assessed by 7-AAD. **p < 0.01 and ***p < 0.001, significantly greater than values for single agent treatment. For these and subsequent studies, values represent the means ± S.D. for experiments performed in triplicate on at least 3 separate occasions. **B**. Cells were exposed to volasertib and belinostat as described above alone or together for 48 hr, after which cells were enumerated by hemocytometer (left panel, p < 0.001, significantly greater than values for single agent treatment). SU-DHL8 were exposed to increasing concentrations of volasertib and belinostat, after which cell growth and viability were evaluated using the CellTiter-Glo Luminescent assay (right panel). **C**. SU-DHL4 cells were exposed to the indicated concentration of volasertib in the presence or absence of belinostat for 48 hr, after which cell death was assessed by 7-AAD. *p < 0.05, **p < 0.01, significantly greater than values for single agent treatment. **D**. SU-DHL4 cells were treated with volasertib (25 nmol/L) or belinostat (400 nmol/L) individually or in combination for the indicated intervals, after which the extent of cell death was determined by 7-AAD uptake and flow cytometry. *p < 0.05, ***p < 0.001, significantly greater than values for single agent treatment. **E**. SU-DHL4 cells were treated with a range of volasertib and belinostat concentrations administered at a fixed ratio. At the end of 48 hr, the percentage of cell death was determined by monitoring 7AAD^+^ cells. CI values were determined in relation to the fractional effect by using Calcusyn software. CI values less than 1.0 correspond to synergistic interactions.

To characterize volasertib/belinostat interactions in NHL cells, studies were performed in GC-DLCBL (SU-DHL4, SU-DHL8, SU-DHL16), ABC-DLBCL (U2932, HBL1), and double-hit DLBCL cells (OCI-LY-18, Carnaval). For these studies, very low concentrations of volasertib were used (e.g., 5-30 nM) in conjunction with low and pharmacologically achievable concentrations of belinostat (e.g., 100-400 nM). In all cells, combined exposure (48 hr) resulted in a marked increase in cell death compared to single-agent treatment, reflected by 7-AAD uptake (Figure 1A). Comparable results were obtained when loss of survival was monitored by the CellTiter-Glo MTT assay (Figure 1B, left panel), visualized for SU-DHL8 cells in Figure 1B, right panel.

Volasertib dose-response studies in GC-DLBCL SU-DHL4 cells revealed that concentrations as low as 12 nM significantly increased the lethal effects of 300-500 nM belinostat, with higher concentrations (e.g., 25 nM) sharply increasing cell death (Figure 1C). Time course studies showed significant increases in cell death for the combination as early as after 24 hr of combined drug exposure, which increased progressively over the ensuing 24 hr (Figure 1D). Median Dose Effect analysis, in which agents were administered over a range of concentrations at a fixed ratio yielded CI (combination index) values considerably less than 1.0, indicating a synergistic interaction. Very similar patterns of drug interactions were observed in double-hit DLBCL cells (OCI-LY-18; Supplementary Figure 1); other GC-DLBCL cells (SU-DHL8, SU-DHL16; Supplementary Figure 2); and ABC-DLBCL cells (HBL-1, U2932; Supplementary Figure 3).

### The volasertib/belinostat regimen is active against MCL, bortezomib-resistant DLBCL cells, and primary NHL cells but not against normal hematopoietic progenitors

**Figure 2:**
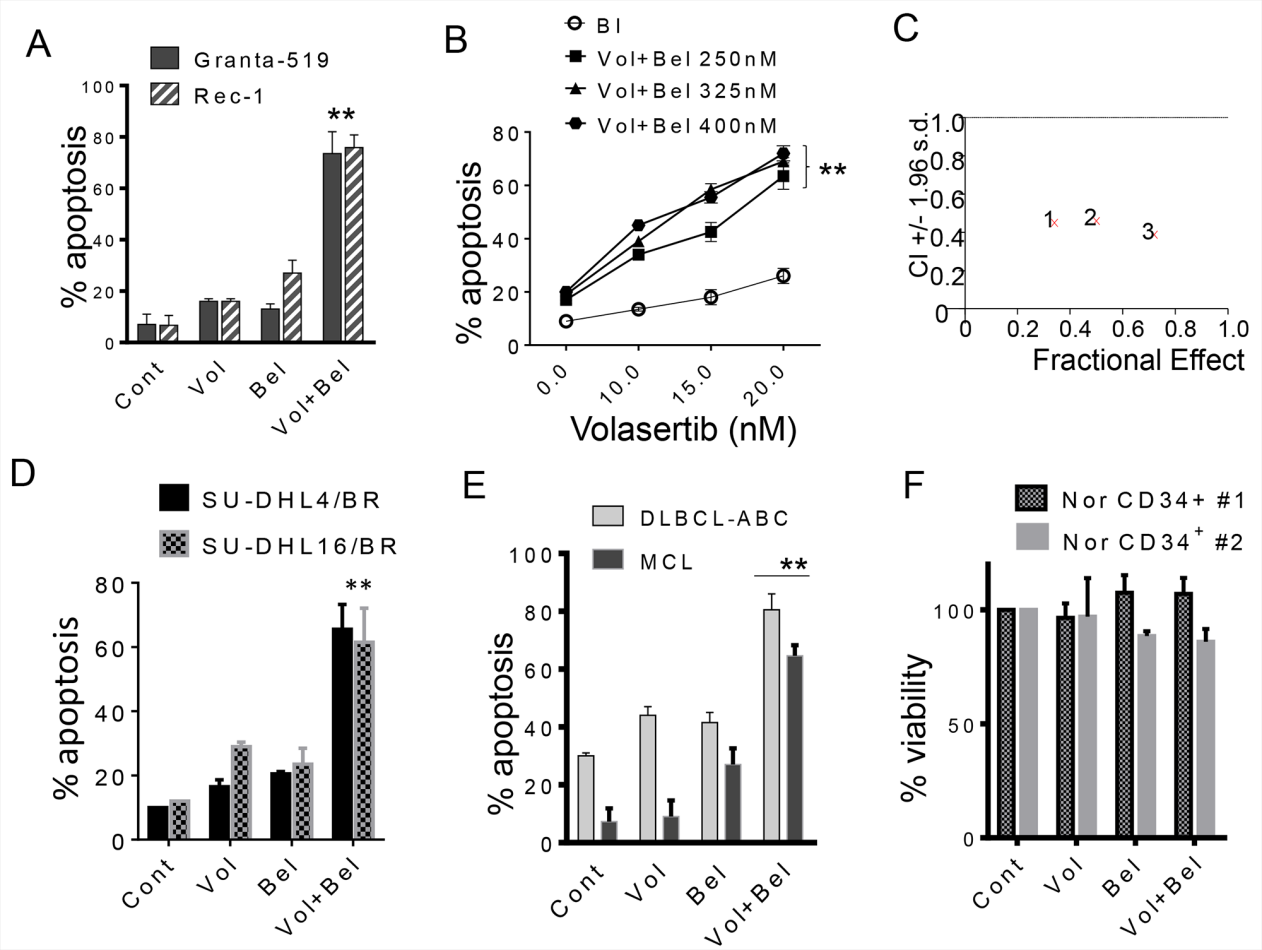
Co-treatment with volasertib and belinostat synergistically induces cell death in mantle cell lymphoma, bortezomib-resistant DLBCL cells, and primary patient specimens, but not normal CD34^+^ bone marrow cells **A**. Granta-519 and Rec-1 (MCL) cells were exposed to volasertib (20-30 nmol/L) and belinostat (200-300 nmol/L) alone or in combination for 48 hr, after which cell death was assessed by 7-AAD. **p < 0.01, significantly greater than values for single agent treatment. **B**. Granta-519 cells were exposed to the indicated concentration of volasertib in the presence or absence of belinostat for 48 hours after which cell death was assessed by 7-AAD. **p < 0.01, significantly greater than values for single agent treatment. **C**. Granta-519 cells were treated with a range of volasertib and belinostat concentrations. At the end of this period, the percentage of 7AAD^+^ cells was determined by flow cytometry. CI values less than 1.0 reflect synergistic interactions. **D**. SU-DHL4/BR and SU-DHL16/BR cells were exposed to volasertib (5-30 nmol/L) and belinostat (100-400 nmol/L) alone or together for 48 hr, after which cell death was assessed by 7-AAD. **E**. Mononuclear peripheral blood cells from a MCL patient in leukemic phase and BM cells from a DLBCL patient with bone marrow infiltration (> 65% lymphoma cells in each case) were exposed to volasertib (15 nmol/L) or belinostat (200-300 nmol/L) individually in combination for 48 hours, after which the percentage of apoptotic cells was determined by annexin V/PI (**p < 0.01, significantly greater than values for single-agent treatment). **F**. Mononuclear cord blood cells (two separate experiments) were isolated and exposed to volasertib (25 nmol/L) or belinostat 250 nmol/L individually or in combination for 48 hours, after which viable (non-apoptotic) CD34^+^ cells was determined by annexin V/PI positivity. P values for the combination were > 0.05, not significantly different compared to values for either agent alone.

Parallel studies were performed in MCL cell lines. Exposure (48 hr) of Rec-1 and Granta MCL cells to minimally toxic concentrations of volasertib (e.g. 20 nM) and belinostat (300 nM) in combination resulted in a marked increase in cell death (Figure 2A). Dose response and Median Dose Effect analysis yielded results very similar to those seen in the case of DLBCL cells (Figure 2B and 2C).

Proteasome inhibitors such as bortezomib have shown activity certain types of lymphoma (e.g., mantle cell lymphoma) [38, 39] and possibly the ABC-DLBCL sub-type [40, 41]. To determine whether the volasertib/belinostat regimen maintained activity in the presence of bortezomib resistance, studies were performed in DLBCL (SUDHL-4 and SUDHL-16) rendered highly resistant to bortezomib by continuous culture in bortezomib (Supplementary Figure 4A). These cells exhibited up-regulation of the proteasome sub-unit PSMB5 as well as mutation of the bortezomib binding site (C235→Ala49Val), resulting in conformational changes to the bortezomib-binding pocket of this subunit [7] (Supplementary Figure 4). These bortezomib-resistant cells remained fully sensitive to the volasertib/belinostat regimen (Figure 2D). Studies were then extended to include two primary specimens, including a MCL and an ABC-DLBCL sample. In each case, combining volasertib (15-20 nM) with belinostat (200-300 nM) resulted in significant increases in cell death compared to individual exposure (Figure 2E). Similar results were obtained in bortezomib-resistant MCL cells (data not shown). Notably, identical exposures (e.g., volasertib 25 nM; belinostat 250 nM) had a negligible effect on normal CD34^+^ hematopoietic cells (Figure 2F).

### Combined volasertib/belinostat exposure results in pronounced mitotic arrest, increased phospho-histone H3 accumulation, a marked increase in mitotic errors, and mitotic cell death

**Figure 3:**
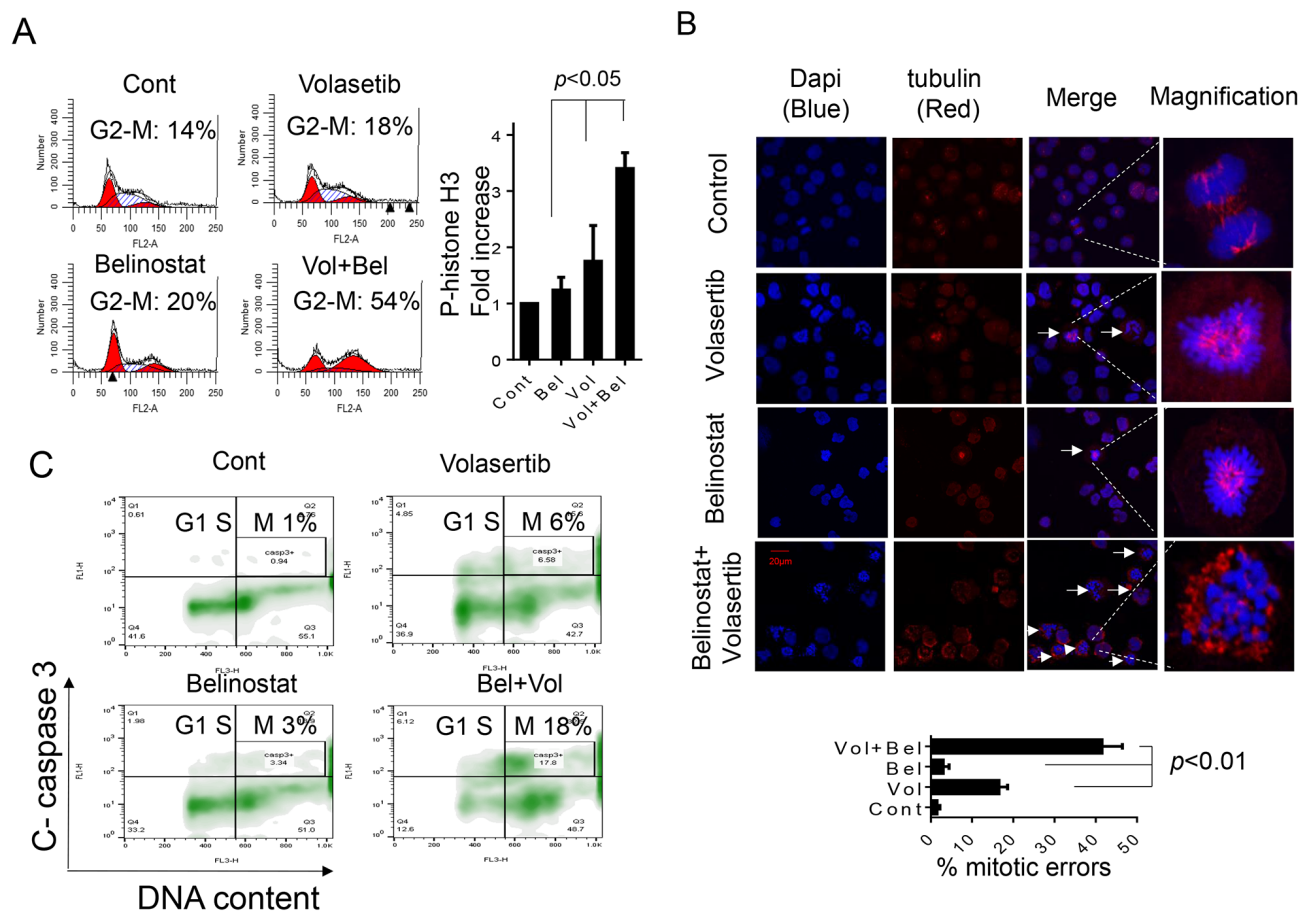
The volasertib/belinostat regimen induces mitotic arrest, frequent mitotic errors and mitotic catastrophe in DLBCL cells **A**. SU-DHL4 cells were exposed to 400 nM belinostat and 25 nM volasertib alone or in combination for 30 hr, after which cells were fixed and cell cycle distribution analyzed by flow cytometry (left panel). Cells treated as above were fixed and stained with p-Histone H3. The percentage of p-Histone H3-positive cells was then compared to values for single-agent treatment or untreated controls. (Values indicate fold increases compared to controls; right panel, p < 0.05). **B**. SU-DHL4 cells were treated as above for 30 hr and immunofluorescence staining performed utilizing antibody directed against α-tubulin (red) and DNA counterstained with DAPI (blue). Arrows indicate cells displaying mitotic errors. High magnification images of a representative cell in each treatment group are shown in the right panels. A total of 100 cells per treatment were enumerated as normal or displaying mitotic errors including multi-polar spindles, improper tubulin alignment, or defective cytokinesis. The percentage of mitotic errors cells was determined and displayed in the lower panel with values for combined treatment significantly greater than those for control or single-agent treatment; p < 0.01. **C**. The distribution of apoptotic SU-DHL4 cells in various cell-cycle phases following treatment with volasertib ± belinostat as above was then determined by combining staining for cleaved caspase-3 and DNA content (PI). Representative results are shown indicating caspase-3 activation in specific cell-cycle phases, including G_2_M.

In view of evidence that both PLK1 and HDAC inhibitors disrupt mitosis [12, 28, 29, 42, 43], the effects of volasertib and belinostat, administered alone or in combination, were examined on mitotic events in SU-DHL4 cells. Exposure of SU-DHL4 cells to volasertib or belinostat individually resulted in only modest arrest of cells in G_2_M (Figure 3A, left panels), and moderately increased phospho-histone H3 expression (Figure 3A, right panel). However, combined exposure resulted in a marked accumulation of cells in M-phase (~54%), and a dramatic increase in phospho-histone H3. Similar results were obtained in HBL1 and SUDHL16 (Supplementary Figure 6 and data not shown).

To investigate further the impact of the agents on aberrant mitosis, control and treated cells were co-stained with DAPI (blue) and α-tubulin (red) to visualize chromosomes/DNA and mitotic spindles respectively. Both volasertib or belinostat alone induced mitotic errors (e.g., mono-polar spindles, abnormal mitotic spindles, improper tubulin alignment, defective cytokinesis etc.) in a limited number of cells (Figure 3B, arrows). However, combined treatment resulted in a dramatic increase in aberrant mitotic events characterized by a high percentage of cells arrested in prometaphase exhibiting the virtual absence of recognizable mitotic spindles accompanied by marked chromosome condensation. As shown in Figure 3B (lower panel), the total number of mitotic errors in the combined treatment group was significantly higher than that observed with either volasertib or belinostat alone (*p* < 0.01). Finally, flow cytometric analysis of cells co-stained with PI and activated caspase-3 demonstrated that individual treatment with volasertib or belinostat alone minimally increased the percentage of apoptotic cells in M-phase (e.g., 6% and 3% for volasertib and belinostat respectively), whereas combined treatment resulted in a marked increase in cell death in this phase (e.g., 18%; Figure 3C), compatible with mitotic catastrophe. Similar results were observed in HBL1 cells (Supplementary Figure 6). Together, these findings indicate that combined volasertib/belinostat treatment of DLBCL cells induces mitotic arrest, frequent mitotic aberrations, and M-phase cell death.

### Combined exposure of DLBCL cells to volasertib and belinostat results in caspase activation, DNA damage, and marked c-Myc down-regulation

**Figure 4:**
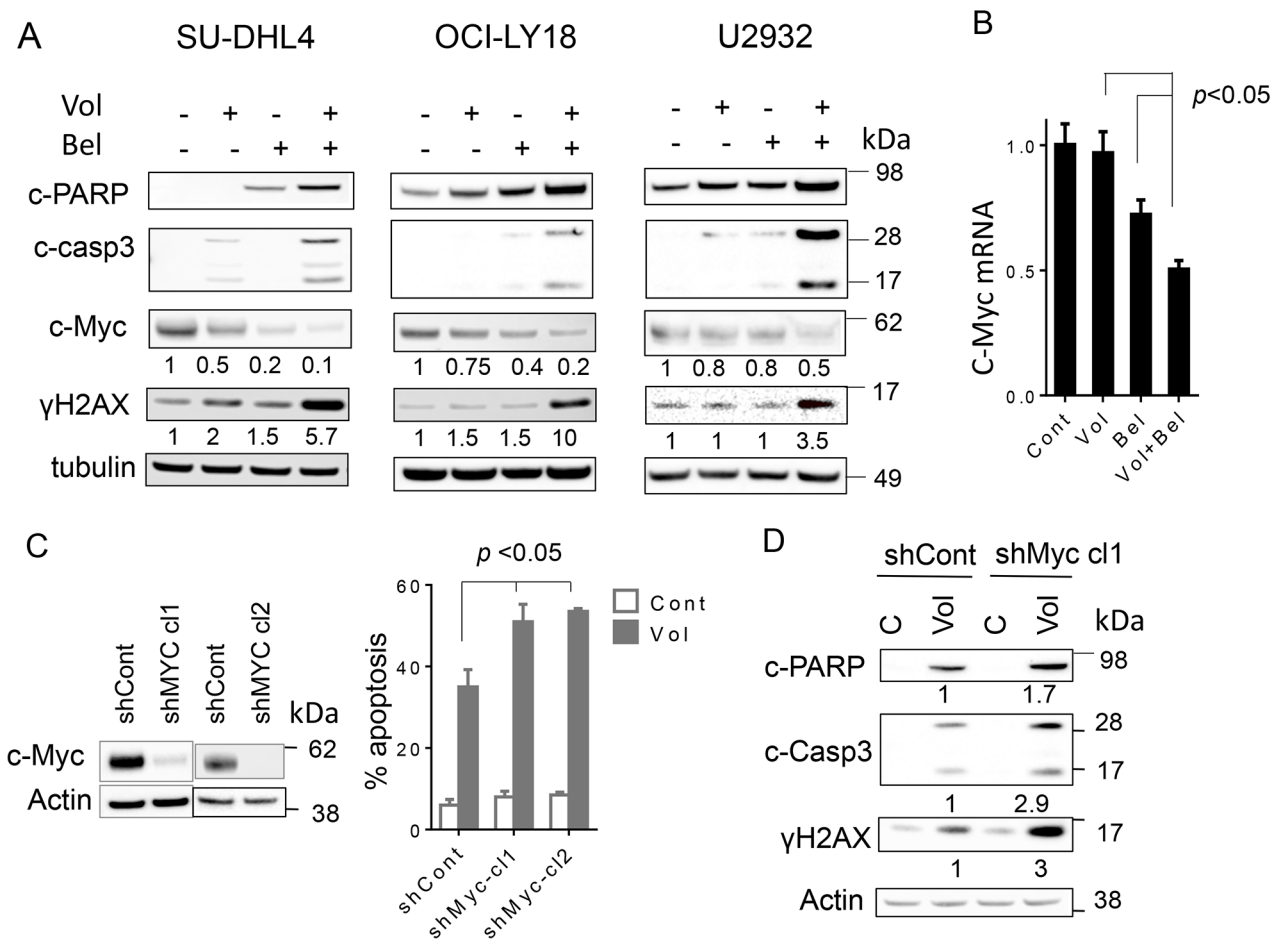
Co-exposure of DLBCL to volasertib and belinostat leads to induction DNA damage, downregulates c-Myc and knocking down of c-Myc potentiates the lethality of volasertib **A**. SU-DHL4, OCI-Ly18 and U2932 cells were treated with volasertib (10-25nM) alone or with belinostat (200-400 nmol/L) for 24 hr after which cells were lysed and proteins extracted. Expression of the indicated proteins was determined by Western blotting using the indicated antibodies. Each lane was loaded with 25 μg of protein; blots were stripped and re-probed with tubulin to ensure equivalent loading and transfer. Results are representative of three replicate experiments. Numbers under the blots correspond to densitometric values normalized to controls arbitrarily set to 1.0. **B**. SU-DHL4 cells were exposed to volasertib (25 nmol/L) ± belinostat (400 nmol/L) for 24 hr after which mRNA of c-Myc was extracted and quantified as described in methods. (p < 0.05, significantly less than values for single-agent treatment). **C**. c-Myc shRNA (shc-Myc clone1 and clone2) and scrambled-sequence control shRNA (shCont) SU-DHL4 cells were generated and the cells were treated with volasertib (25 nmol/L) for 48 hr, after which the percentage of dead cells was determined by 7-AAD (right panel), (p < 0.05 versus control). **D**. shc-Myc and shCont SU-DHL4 cells were treated with volasertib for 24 hr, after which Western blot analysis was performed to monitor c-PARP, cleaved caspase-3, and γH2A.X expression.

Consistent with effects on cell death, combined exposure of GC-DLBCL cells (SUDHL4), double-hit DLBCL cells (OCI-Ly18), and ABC-DLBCL cells (U2932) resulted in enhanced cleavage of PARP and caspase-3, accompanied by an increase in DNA damage, reflected by increased accumulation of γH2A.X (Figure 4A). In addition, exposure of each cell type to belinostat, particularly when combined with volasertib, resulted in a marked reduction in c-Myc protein expression and mRNA expression (Figure 4B).

As c-Myc deregulation has been implicated in lymphomagenesis [44], attempts were made to determine the functional significance of c-Myc down-regulation by the volasertib/belinostat regimen. To this end, c-Myc was knocked down by shRNA in SU-DHL4 cells, and two clones (SUDHL4-cl1 and -cl2) isolated (Figure 4C, left panels). Both clones were significantly more sensitive to volasertib-mediated cell death than their scrambled-vector counterparts (*p* < 0.05; Figure 4C, right panel). Consistent with these results, c-Myc knock-down increased volasertib-mediated PARP cleavage, caspase-3 activation, and increased γH2A.X formation (Figure 4D). Together, these findings argue that c-Myc down-regulation plays a functional role in volasertib/belinostat lethality in DLBCL cells.

### PLK1 knock-down potentiates belinostat-induced mitotic arrest and lethality

**Figure 5:**
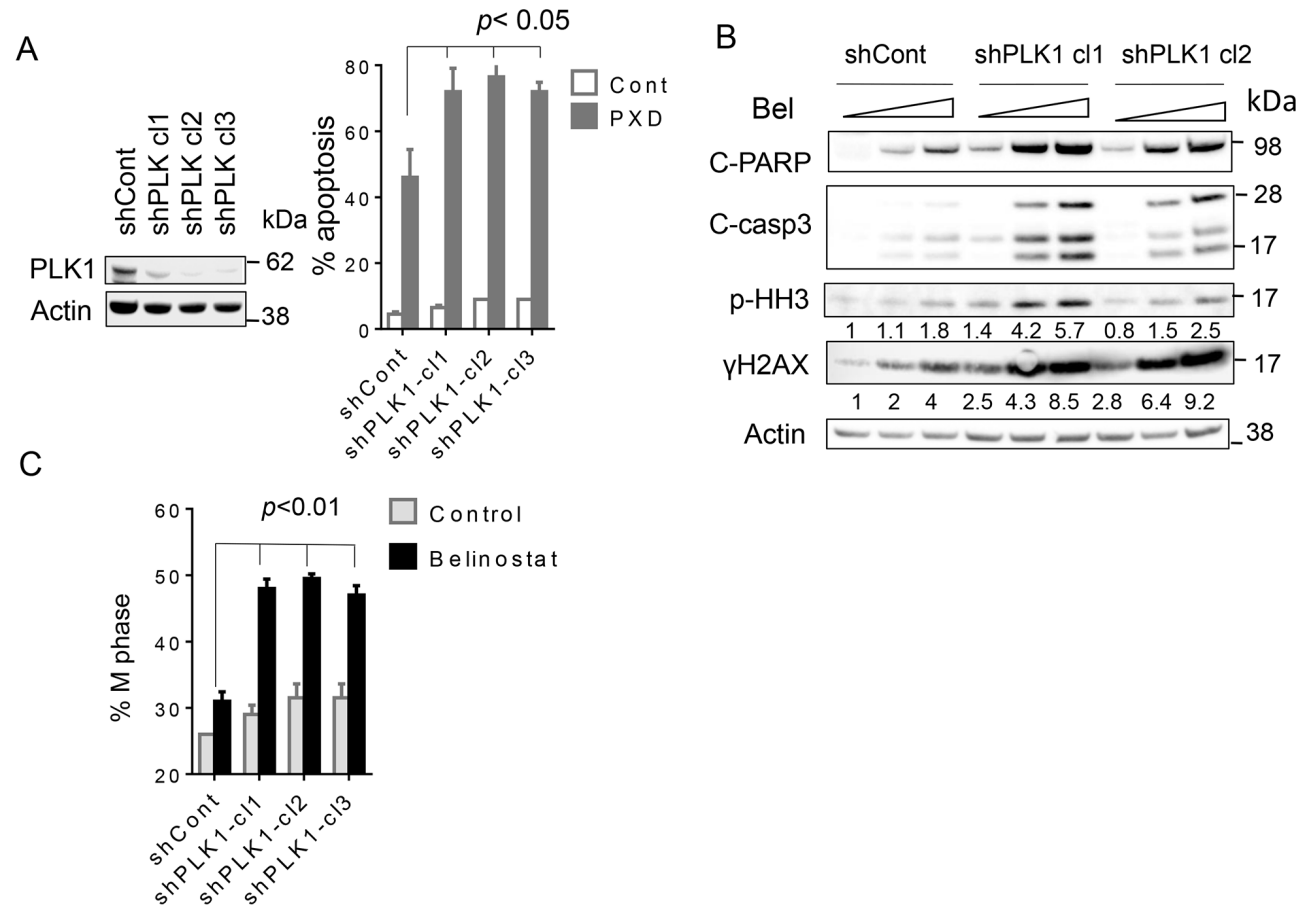
Knockdown of PLK1 strikingly potentiates belinostat-mediated apoptosis in SU-DHL4 cells **A**. SU-DHL4 cells were transfected with shPLK1 or scrambled sequence shRNA (shControl). Three knockdown PLK1 clones were selected (shPLK1 clones1-3) (left panel), the cells exposed to 450 nmol/L of belinostat for 48 hr, after which cell death was monitored by 7-AAD staining (right panel). **B**. shPLK1 and shCont cells were treated with 300 and 450 nmol/L of belinostat for 24 hr, after which Western blot analysis was performed to monitor c-PARP, cleaved caspase-3, p-Histone H3 and γH2A.X expression. Numbers under the blots correspond to densitometric values normalized to actin arbitrarily set to 1.0. **C**. shPLK1 and shCont cells were treated with 450 nmol/L of belinostat for 40 hr after which cell cycle analysis was performed by flow cytometry and the percentage of M cells was determined (p < 0.01 for knock-down cells versus controls).

To address the functional significance of PLK1 disruption in volasertib/belinostat interactions, three SU-DHL4 clones stably expressing PLK1shRNA (shPLK1 clones 1-3) were generated (Figure 5A, left panel). Notably, the PLK1 knockdown clones were significantly more sensitive to belinostat lethality (300-450nM; 48 hr) compared to controls (scrambled sequence-vector) (Figure 5A, right panel; *p* < 0.05 in each case). Consistent with these findings, PLK1shRNA cells exposed to belinostat exhibited increased PARP and caspase-3 cleavage, γH2A.X formation, and phospho-histone H3 induction compared to controls (Figure 5B). Very similar results were obtained in HBL1 cells (Supplementary Figure 7). Cell cycle analysis revealed that belinostat minimally increased the M-phase fraction of scrambled-vector controls, but substantially increased this sub-population in PLK1shRNA cells. Quantitation of results demonstrated very significant increases in belinostat-mediated M-phase arrest in PLK1shRNA clones compared to controls (Figure 5C; p < 0.01).

### The volasertib/belinostat regimen is active *in vivo* against ABC- and double-hit DLBCL cells

**Figure 6:**
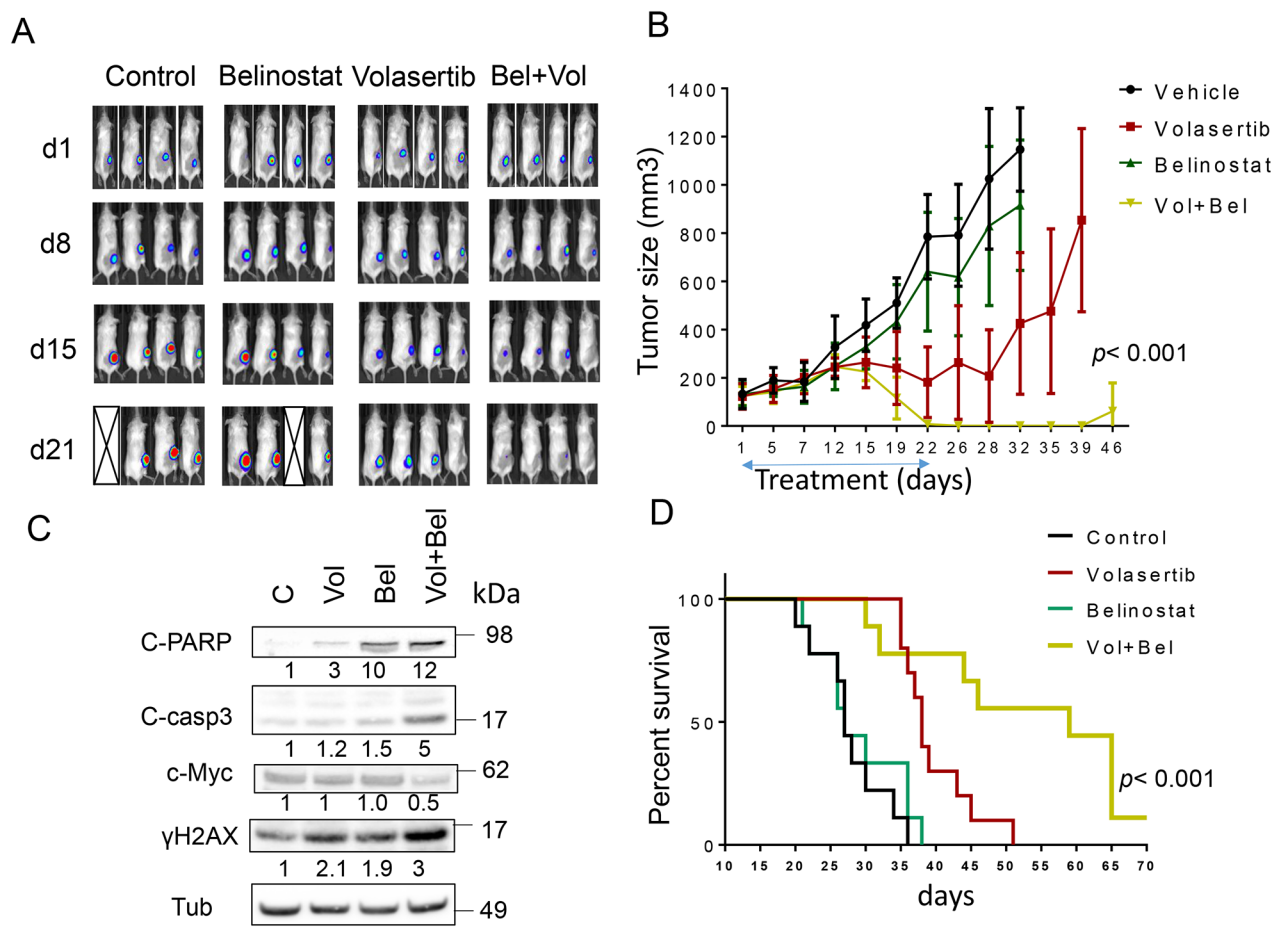
Co-treatment with volasertib and belinostat suppresses tumor growth in a murine xenograft model and prolongs animal survival NOD/SCID-γ mice were subcutaneously inoculated in the right rear flank with 10 × 10^6^ U2932/Luc cells which stably express luciferase. Studies involved 9-10 mice per group. Treatment was initiated after the tumor were visualized, measured, and randomly grouped 10 days after injection of tumor cells. Belinostat was administrated at a dose of 80 mg/kg by i.p 5 days a week. Volasertib was administered at a dose of 12 mg/kg i.p once a week. Control animals were administered equal volumes of vehicle. **A**. Tumor growth was monitored twice weekly by injection of luciferin and imaged by the IVIS 200 imaging system. d=day, empty boxes represent deceased mice. **B**. Tumor size was measured by caliper twice weekly. Tumor volumes were calculated (length × width^2^/2) and plotted against days of treatment. The combination group exhibited significantly smaller tumor size than either single-agent volasertib or belinostat treatment (one-way Anova, *p* < 0.001). **C**. Mice were treated for 14 days, and after tumors reached 1 cm in diameter, a representative mouse in each group was sacrificed. Tumors were resected, homogenized and subjected to Western blot analysis for c-PARP, cleaved caspase-3, p-histone H3 and γH2A.X expression. **D**. Kaplan–Meier analysis was performed to analyze survival of animals. The survival of mice treated with the combination was significantly prolonged compared to mice treated with single agents (p< 0.001). Treatment was discontinued after day 21.

To evaluate the *in vivo* implications of these findings, flank and systemic DLBCL xenograft models were employed. For the former, 10 × 10^6^ luciferase-labeled U2932 ABC-DLBCL cells were inoculated in the flanks of NOD/SCID-γ mice, and after 10 days, animals were randomly divided into 4 groups. They were then treated with belinostat (80 mg/kg ip 5 d/wk) ± volasertib (12 mg/kg ip weekly) after which tumor growth was monitored by luciferase imaging as well as caliper-based tumor size measurements. As shown in Figure 6A, imaging of mice after luciferin injection revealed that control and belinostat-treated animals exhibited robust tumor growth after 21 days with several animals dying. Volasertib treatment alone modestly reduced tumor growth compared to control and belinostat-treated animals. However, combined treatment sharply reduced tumor growth, reflected by marked attenuation of luciferase signal over this interval. Consistent results were obtained when tumor size was monitored (Figure 6B). Whereas belinostat alone had little effect on tumor growth, volasertib slightly reduced mean tumor size after 2-3 weeks of treatment. However, combined treatment dramatically reduced tumor growth to nearly undetectable levels after 22 days of treatment (*p*< 0.001; one-way Anova). Toxicity for the combination, reflected by behavioral changes, fur loss, or loss of body weight (Supplementary Figure 8) was minimal. In companion experiments, Western blot analysis of excised tumor tissue revealed more pronounced cleavage of caspase-3 and PARP, c-Myc down-regulation, and γH2A.X formation in tumors obtained from mice treated with both agents (Figure 6C). Finally, Kaplan-Meier analysis revealed a very significant improvement in survival for combined (56.2 ± 14.5 days) *vs*. volasertib (39.7 ± 5.1 days), belinosat (29.1 ± 6.3 days) alone or control (27.8 ± 5.1 days) treatment (*p*< 0.001).

**Figure 7:**
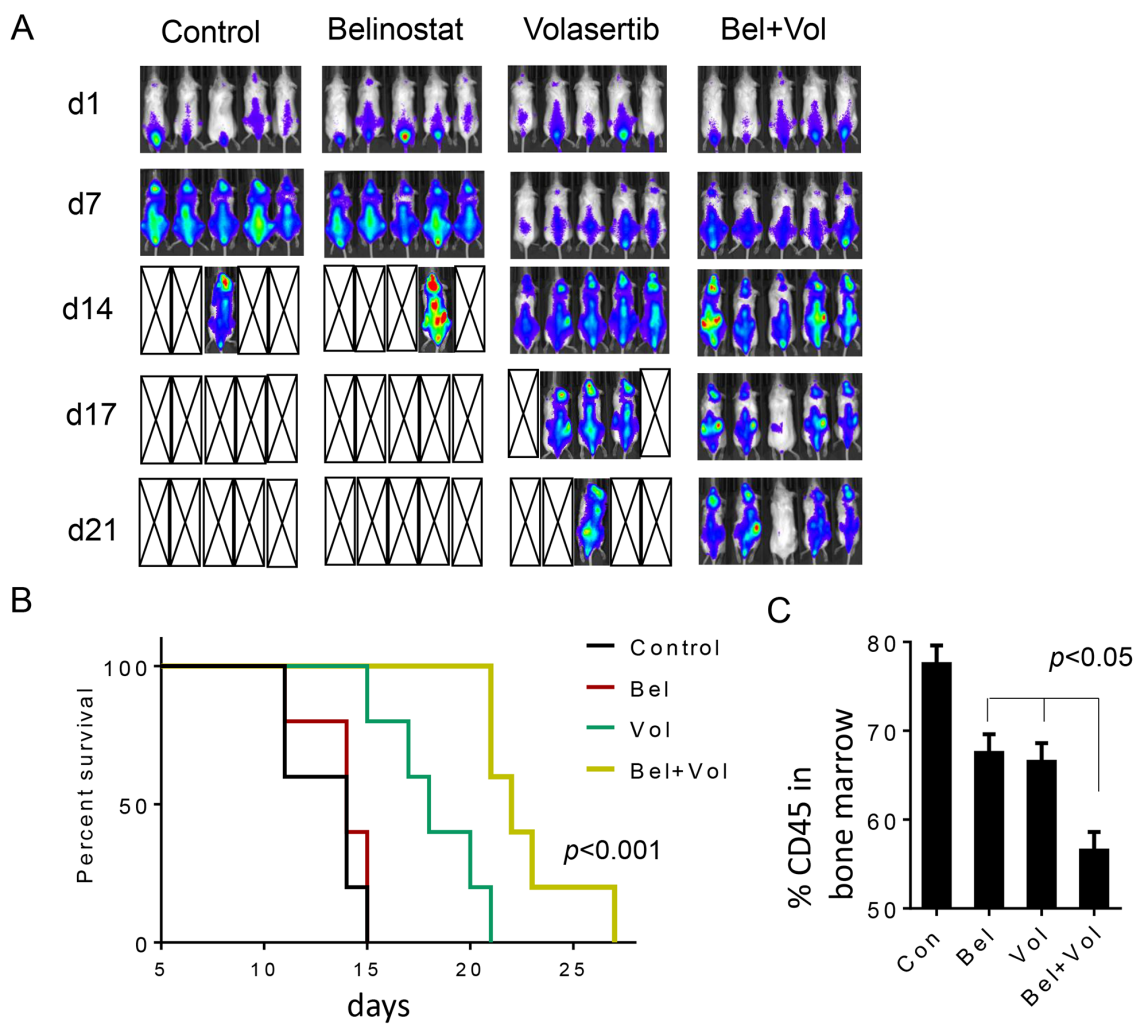
Belinostat potentiates volasertib activity against systemic OCI-Ly18 in a murine xenograft model and prolongs animal survival OCI-Ly18/Luciferase cells were injected into NOD/SCID-gamma mice via tail vein (7.5 × 10^6^ cells). The mice were then injected with firefly luciferin and imaged by IVIS twice weekly. Treatment was initiated after tumors (OCI-Ly18/Luc) were visualized and randomly grouped. Studies involved 5 mice per group. Belinostat was administrated at a dose of 80 mg/kg by intraperitoneal injection (i.p.) 5 days a week. Volasertib was administered at a dose of 12 mg/kg via i.p. injection once a week. Control animals were administered equal volumes of vehicle. Treatment was discontinued after day 21. **A**. Tumor growth was monitored twice weekly by injection luciferin followed by imaging by the IVIS 200 imaging system. d=day, empty boxes represent deceased mice. **B**. Survival of mice treated as above was evaluated from the first day of treatment until death using Kaplan–Meier curves. The survival of mice treated with the combination was significantly prolonged compared to mice treated with single agents, p< 0.001. **C**. Cells from bone marrow obtained from one mouse in each group after 2 weeks of treatment were stained with fluorescently-labeled CD45 antibodies, after which they were analyzed by flow cytometry.

Parallel studies were performed using a systemic double-hit DLBCL model (OCI-Ly18). Five days after iv injection of cells, animals were randomly grouped and treated with volasertib ± belinostat as described above, after which tumors were imaged at the indicated intervals (Figure 7A). Belinostat alone failed to suppress tumor growth, and all animals from both vehicle and belinostat groups were dead after 17 days (average survival days for control: 13 ± 1.87, belinostat 13.8 ± 1.64). Volasertib alone modestly reduced tumor growth, but the majority of animals were dead by 21 days (average survival 18.4 ± 2.61 days). However, while the combination failed to eradicate systemic tumor in most animals, all animals were alive at this interval (average survival 22.8 ± 2.95 days; Figure 7B). Overall survival was significantly greater for combined treatment versus volasertib or belinostat treatment alone (*p*< 0.001). Finally, in a separate study, animals were sacrificed after 10 days of treatment, after which marrows were extracted and expression of the human marker CD45 monitored by flow cytometry. Combined treatment significantly reduced bone marrow CD45+ cells compared to single-agent treatment (Figure 7C; *p*< 0.05).

## DISCUSSION

Evidence that PLK1 are over-expressed in neoplastic cells, including NHL cells, but not in normal cells [19] has stimulated interest in agents such as volasertib in the treatment of lymphoma [18, 37, 45]. In fact, 5 out of 6 cell lines we investigated exhibited high PLK1 expression compared to normal CD19+ cells (Supplementary Figure 5). Pre-clinical studies suggest that lymphoma cells, particularly those of T-cell origin, may be particularly vulnerable to such agents [45], and the PLK1 inhibitor MLN0905 has shown *in vivo* activity in DLBCL xenograft models [20]. Moreover, the observation that in a phase I trial of the volasertib precursor, BI 2536, objective responses in NHL were observed [46] further supports this strategy. Similarly, aberrant HDAC expression in lymphoma [47] argues for the use of HDACIs in this disorder, and in fact these agents have received approval or orphan drug status in CTCL/PTCL and some forms of DLBCL [48]. Furthermore, the observation that PLK1 and HDAC play key roles in the DDR [8, 27, 35] raises the possibility that PLK1 and HDAC inhibitors may cooperate to trigger DNA damage in neoplastic cells. In this context, previous studies have shown that such agents interact to potentiate apoptosis in prostate cancer cells [49]. The present results indicate that the clinically relevant PLK1 inhibitor volasertib interacts synergistically with the approved HDACI belinostat reduce tumor growth in diverse NHL cell types both *in vitro* and *in vivo*, and suggest that the mechanisms underlying this phenomenon are likely to be multifactorial.

The mechanism of cell death induced by volasertib/belinostat co-administration appears to involve one or more forms of mitotic cell death/mitotic catastrophe. For example, PLK1 inhibitors induce multiple mitotic errors in CTCL cells, including the formation of monopolar or multi-polar cells, and a failure of cytokinesis [43]. In addition, the PLK1 inhibitor BI 2536 triggers mitotic catastrophe in non-small cell lung cancer cells through activation of the spindle assembly checkpoint [10]. In this regard, DNA-damaging agents characteristically induce mitotic arrest, allowing cells to repair the damage if it is not extensive, or undergo apoptosis if it is in order to preserve genomic integrity [50]. Consequently, PLK1 inhibitors may enhance the activity of genotoxic agents by disrupting the latter process [5, 9]. Against this background, attention has recently focused on the ability of HDACIs, in addition to their epigenetic actions, to kill tumor cells through induction of DNA damage [36, 51], a phenomenon which involves multiple mechanisms, including induction of ROS, disruption of DNA damage checkpoints (e.g., the G2 checkpoint, and interference with homologous recombination or non-homologous end-joining DNA repair) [27, 30, 42]. Of note, HDACIs have also been shown to trigger mitotic catastrophe, e.g., in glioma cells, by abrogating the G2 checkpoint [42, 52]. It is therefore tempting to speculate that the enhanced cell death observed in cells co-exposed to volasertib and belinostat reflects, at least in part, potentiation of the lethal consequences of belinostat-induced DNA damage by disruption of the mitotic checkpoint apparatus e.g., by volasertib. Furthermore, this process may be amplified by direct interference in the DDR (e.g., checkpoints and DNA repair) by belinostat itself. In accord with this notion, cells exposed to volasertib in combination with belinostat exhibited massive G_2_M arrest, a dramatic increase in mitotic errors, and a pronounced increase in the DNA damage marker γH2A.X. The finding that PLK1 shRNA knock-down cells displayed similar events when exposed to belinostat provides additional support for this concept, and argues for on-target effects.

Both PLK1 and HDAC inhibitors administered individually have been shown to induce mitotic slippage [12, 29]. Mitotic slippage occurs as a consequence of failure of the mitotic checkpoint spindle, resulting in failed cytokinesis and inappropriate mitotic exit, culminating in the formation of polyploid cells. Based on these considerations, it seems plausible that potentiation of this process might contribute to volasertib/belinostat synergism. However, we were unable to document enhanced slippage in DLBCL cells co-exposed to volasertib and belinostat (Nguyen and Grant, unpublished observations). One possible explanation for this finding is that the pronounced increase in HDACI-mediated DNA damage observed with co-treatment, coupled with disruption of the DDR, particularly the mitotic checkpoint and DNA repair, results in a cell death signal in mitosis. Consistent with this hypothesis, apoptosis induced by the volasertib/belinostat regimen predominantly occurred in the M-phase cell population (Figure 3). Marked induction of mitotic cell death by this mechanism would in all likelihood prevent the emergence of multi-nucleated cells. Another possible explanation is that the low concentrations of volasertib used in these studies may not be sufficient to induce mitotic slippage observed with higher PLK1 inhibitor concentrations [12]. Additional studies will be necessary to confirm or refute these hypotheses.

It is noteworthy that combined exposure to volasertib and belinostat induced pronounced down-regulation of c-Myc, an oncogene that plays a key role in lymphomagenesis [53]. HDACIs are known to down-regulate c-Myc in tumor cells, including lymphoma cells, through a transcriptional inhibitory mechanism [54]. Interestingly, PLK1 inhibitors can mimic the actions of bromodomain inhibitors (BET) [55], which down-regulate diverse genes, including c-Myc through transcriptional repression and interference with chain initiation [56, 57]. However, volasertib, administered at the low nM concentrations used here minimally down-regulated c-Myc message or protein, whereas belinostat alone significantly down-regulated both. These findings argue that volasertib, through a yet to be determined mechanism, potentiates down-regulation of c-Myc transcription by belinostat. The ability of c-Myc shRNA knock-down to recapitulate the effects of belinostat and to increase volasertib lethality (Figure 4B) argues that this phenomenon plays a significant functional role in the anti-lymphoma activity of this regimen. Furthermore, evidence that c-Myc represents a major determinant of mitotic cell fate [58] raises the possibility that down-regulation of c-Myc in NHL cells by the volasertib/belinostat regimen contributes to the pronounced increase in mitotic cell death.

The volasertib/belinostat regimen exhibited *in vitro* activity not only against GC-DLBCL, which is associated with a favorable prognosis [59], but also against its ABC-DLBCL counterpart. ABC-DLBCL is characterized by a dependence upon NF-κB for survival [60], and a generally inferior prognosis [61]. Very similar results were obtained in the case of mantle cell lymphoma, another aggressive form of NHL. Importantly, the combination was also effective against “double-hit” lymphoma cell lines which overexpress both c-Myc and Bcl-2. Double-hit lymphomas, as well as their counterparts over-expressing Bcl-6, have a particularly poor prognosis, and respond poorly to standard and investigational therapies [62]. In contrast to its toxicity toward NHL cells, the volasertib/belinostat regimen was minimally toxic to normal hematopoietic progenitors, raising the possibility of therapeutic selectivity. Of note, HDACIs have been shown to kill neoplastic cells preferentially compared to normal cells [36]. This phenomenon may reflect the selective ability of normal versus neoplastic cells to mount a DNA damage response in response to HDACIs, particularly the up-regulation of DNA repair proteins [36]. As previously discussed, transformed cells exhibit higher basal expression of PLK1 than normal cells [14], arguing that the former may be more dependent upon this mitotic kinase. Thus, combination of these agents may have the net effect of amplifying the postulated selectivity of the individual agents.

The volasertib/belinostat regimen was well tolerated in two DLBCL models: an ABC flank and a systemic double-hit (OCI-Ly18) model, findings consistent with the relatively minimal toxicity of the regimen toward normal hematopoietic progenitors (Figure 2F). In each case, survival of mice receiving both agents was significantly greater than that observed in mice receiving the agents individually. In the former case, bone marrow infiltration by lymphoma cells was also significantly reduced. Importantly, several of the pharmacodynamic markers observed *in vitro* were recapitulated in the *in vivo* setting e.g., a reduction in tumor cell c-Myc expression accompanied by an increase in DNA damage (e.g., up-regulation of γH2A.X, arguing that similar mechanisms underlying interactions may be operative in the two settings. Whether mechanisms identified and validated *in vitro* play analogous functional roles in the *in vivo* setting remains to be determined, and studies addressing this question are currently underway.

In summary, evidence of PLK1 activity in pre-clinical DLBCL and other NHL models [21, 45], early signals of activity of volasertib in lymphoid malignancies [23], and the present findings demonstrating potentiation of volasertib anti-lymphoma efficacy by HDACIs support the development of a PLK1/HDAC inhibitor regimen in NHL. The bulk of evidence of suggests that multi-factorial mechanisms underlie synergism, including potentiation of DNA damage, disruption of DNA damage checkpoints, particularly the mitotic checkpoint, induction of mitotic cell death, and down-regulation of c-Myc, among others. Based upon the present findings, a phase 1 trial of volasertib (or other PLK1 inhibitors) in combination with an HDACI such as belinostat for patients with relapsed/refractory NHL appears justified. In addition to testing the tolerability of this regimen, and identifying the recommended phase II dose (RP2D), such a trial could help to determine whether post-treatment pharmacodynamic changes in tumor cells can recapitulate those observed pre-clinically. Consequently, plans to develop this strategy further in patients with NHL are currently underway.

## MATERIALS AND METHODS

### Cells

SU-DHL4 (GC subtype) cells were provided by Dr. Lisa Rimsza (University of Arizona). SU-DHL16 (GC subtype), Granta-519 (mantle cell lymphoma-MCL), and OCI-Ly18 and Carnaval (both double-hit lymphoma) were purchased from DSMZ (Braunschweig, Germany). HBL-1, TMD8, and U2932 (all ABC subtype) were kindly provided by Dr. Ari Melnick (Weill-Cornell Medical Center). Rec-1 (MCL) cells were generously provided by Dr. Steven Bernstein (University of Rochester Medical Center). Cell lines were authenticated by DNA profiling [short tandem repeat (STR) analysis] at the University of Arizona Genetics Core using a Promega PowerPlex16HS Assay with15 autosomal loci plus X/Y. All lines were frozen within two months of receipt, and fresh aliquots were thawed before lines reached 6 months in culture.

### Immunoblot and primary antibody

Western blot analysis was carried out as previously described [63]. Primary antibodies used in these studies were: cleaved PARP, cleaved caspase-3, phospho-histone H2A.X (Ser139), PLK1, PSMB5 and c-Myc (Cell Signaling Technology, Danvers, MA), Mcl-1 (BD Biosciences, San Jose, CA), α-tubulin (EMD Millipore, Billerica, MA), actin (Sigma-Aldrich, St. Louis, MO), NOXA (Enzo Life Sciences, Farmingdale, NY).

### Cell growth and viability

Cell growth and viability were monitored by hemocytometer or assessed by the CellTiter-Glo Luminescent Assay (Promega, Madison, WI) and visualized by Li-Cor (Li-Cor Inc, Lincoln, Nebraska).

### Cell cycle analysis

Cell cycle distribution was determined by flow cytometry after fixation and incorporation of propidium iodide (PI). The data were analyzed using a commercial software program (Modfit, Becton Dickinson) as per standard protocol.

### Distribution of apoptotic cells in cell-cycle phases

Caspase-3 activation/DNA content analysis was performed by dual-parameter flow cytometry to monitor apoptotic cells within specific cell-cycle phases. Cells were stained with a 1:100 dilution of Alexa Fluor 488–conjugated anti-cleaved caspase-3 for 1 hour at 4°C. DNA was stained with PI as described as above. In these studies, sub-diploid (late apoptotic) cells, which cannot be related to a specific cell-cycle compartment, were gated out and not included in the analysis.

### DNA sequence of the PSMB5 and qPCR of c-Myc

Total RNA was extracted by using the RNeasy mini Kit (Qiagen, Valencia, CA), followed by reverse transcription into cDNA [63, 64]. Exon II of the *PSMB5* gene was amplified by means of PCR using the following primer set: forward, 5′-TTCCGCCATGGAGTCATA-3′; and reverse, 5′-GTTGGCAAGCAGTTTGGA-3′ [65]. The PCR product was purified and sequenced. Sequence data were aligned and analyzed with Bioedit software. Quantitative PCR of c-Myc was performed in triplicate wells using SensiMix SYBR Hi Rox kit (BIOLINE, TauntonMA) and StepOnePlus™ Real-Time PCR System (Applied Biosystems, Foster City, CA). Amplification of actin B was used as a normalization control to quantify the relative expression. Specific primers for c-Myc (forward 5′- GGACCCGCTTCTCTGAAAGG-3′ and reverse 5′- TAACGTTGAGGGGCATCGTC-3′) and actin (forward 5′- TGACCCAGGACTCTCTTCTCT -3′ and reverse 5′- CTCATGGCCTTGTCACACGA -3′) were used.

### Confocal microscopy

Briefly, cells were fixed with 4% paraformaldehyde and permeabilized with 0.1% Triton X-100. α-tubulin was detected by anti-α-tubulin/Alexa Fluor 555 (EMD Millipore, Billerica, MA). Slides were mounted in mounting medium containing 4′,6-diamidino-2-phenylindole (DAPI; Southern Biotechnology, AL). Slides were analyzed using a Zeiss LSM 700 confocal microscope and software Zen (Carl Zeiss, San Diego, CA).

### Plasmids and transfection

Sh knockdown PLK1 and c-Myc were purchased from Sigma (Cat#SHCLNG08121521MN) and Open System (#RHS4533-NM002467) respectively. Knockdown of PLK1 and c-MYC was accomplished by infecting SU-DHL4 and HBL1 cells with lentiviruses carrying specific shRNA. Luciferase or scrambled shRNA/pLKO.1 was used as control. Lentivirus production was generated using Lipofectamine 3000 (Invitrogen, ThermoFisher Scientific, NJ) following the manufacturer's protocol.

### Reagents

Volasertib (BI 6727) was from Boehringer Ingelheim (Ingelheim, Germany). Belinostat (PXD101) was from Spectrum Pharmaceuticals, Irvine, CA. All agents were formulated in DMSO for *in vitro* use.

### Assessment of apoptosis and flow cytometry

The extent of apoptosis was evaluated by either annexin V–fluorescein isothiocyanate staining (BD Biosciences, MA) or 7-aminoactinomycin D (7-AAD; Sigma-Aldrich, St. Louis, MO) by flow cytometry as described previously [63]. Cleaved caspase-3 Alexa 488 (Cell Signaling, Danvers, MA) and Fc Receptor Blocking Solution (Bio Legend, San Diego, CA), CD45/PE-Cy7 (BD Biosciences, San Jose, CA) were purchased and used as per the manufacturer's protocol.

### Collection and processing of primary normal CD34^+^ and lymphoma patient cells

Normal hematopoietic cells were collected from cord blood with informed consent. Mononuclear cells were isolated by Ficoll-Hypaque gradient separation as described [66]. CD19^+^ or CD34^+^ cells were enriched using a Miltenyi microbead separation system (Miltenyi BioTech, Auburn, CA) according to the manufacturer's protocol. Bone marrow or peripheral blood were collected from patients with DLBCL or MCL, and further enrichment of mononuclear cell populations achieved by Ficoll–Hypaque gradient separation as previously described [66]. These studies have been approved by the Investigational Review Board of Virginia Commonwealth University.

### *In vivo* studies

Animal studies were conducted under an approved protocol by the Virginia Commonwealth University Institutional Animal Care and Use Committee. For flank experiments, female NOD/SCID-gamma (Jackson laboratories) were inoculated subcutaneously in the flank with 10 × 10^6^ luciferase-expressing U2932 cells. Once tumors became apparent (~10 days), tumor volume was measured 2 times per week with calipers using the following formula: tumor volume (mm^3^) = length (mm) × width (mm)^2^/2. For systemic experiments, female NOD/SCID-gamma (Jackson laboratories) were injected intravenously via tail vein with 7.5×10^6^ luciferase-expressing OCI-LY18 cells. The mice were monitored using the IVIS 200 imaging system (Xenogen Corporation, Alameda, CA). Luciferase signals were detected approximately four days after injection. Lymphoma cell growth was monitored by measuring tumor size by caliper or utilizing the imaging system. For both flank and systemic experiments, mice were randomly separated into 4 groups, and each group was treated with vehicle, 12mg/kg volasertib (i.p., once a week for 3 weeks), 80mg/kg belinosat (i.p., 5 days a week for 3 weeks), or the combination of volasertib and volasertib. For *in vivo* studies, volasertib was dissolved in 0.1N HCL as a stock solution and further diluted in 0.9% NaCl for use. Belinostat was dissolved with equivalent amounts of L-arginine in distilled water and sonication.

### Statistical analysis

All results are expressed as the means ±SD. The significance of differences between experimental conditions was determined using two-tailed Student's t test, one-way Anova with post hoc Tukey's multiple comparisons test. The significance of p values was < 0.05 (*), < 0.01 (**) and < 0.001 (***) wherever indicated. Survival rates were analyzed by Kaplan–Meier [67] using Prism 6 (GraphPad Software, CA). Synergistic interactions were defined using Median Dose Effect analysis [68] in conjunction with a commercially available software program (CalcuSyn, Biosoft, Ferguson, MO).

## SUPPLEMENTARY MATERIALS FIGURES



## Abbreviations

DLBCL: Diffuse large B-cell lymphoma
MCL: mantle cell lymphoma
PLK: polo-like kinase
HDACI: histone deacetylase inhibitor
