# Correction: Synergistic interactions between PLK1 and HDAC inhibitors in non-Hodgkin’s lymphoma cells occur *in vitro* and *in vivo* and proceed through multiple mechanisms

**DOI:** 10.18632/oncotarget.27175

**Published:** 2019-09-03

**Authors:** Tri Nguyen, Rebecca Parker, Elisa Hawkins, Beata Holkova, Victor Yazbeck, Akhil Kolluri, Maciej Kmieciak, Mohamed Rahmani, Steven Grant

**Affiliations:** ^1^ Division of Hematology/Oncology, Department of Internal Medicine, Virginia Commonwealth University and the Massey Cancer Center, Richmond, VA, USA; ^2^ Departments of Biochemistry, Virginia Commonwealth University, Richmond, VA, USA; ^3^ Departments of Pharmacology, Virginia Commonwealth University, Richmond, VA, USA; ^4^ Virginia Institute for Molecular Medicine, Virginia Commonwealth University, Richmond, VA, USA; ^5^ Massey Cancer Center, Virginia Commonwealth University Health Sciences Center, Richmond, VA, USA

**This article has been corrected:** Due to errors in image assembly, the IVIS image for the control mouse (panel 3) was inadvertently duplicated as the volasertib-treated mouse (panel 2) at time 0 (d1) in Figure 6A. After reviewing the original files, the correct image for the volasertib-treated time 0 (d1) mouse (panel 2) was located. The corrected Figure 6A is shown below. The authors declare that these corrections do not change the results or conclusions of this paper.

Original article: Oncotarget. 2017; 8:31478–31493. 31478-31493. https://doi.org/10.18632/oncotarget.15649

**Figure 6:**
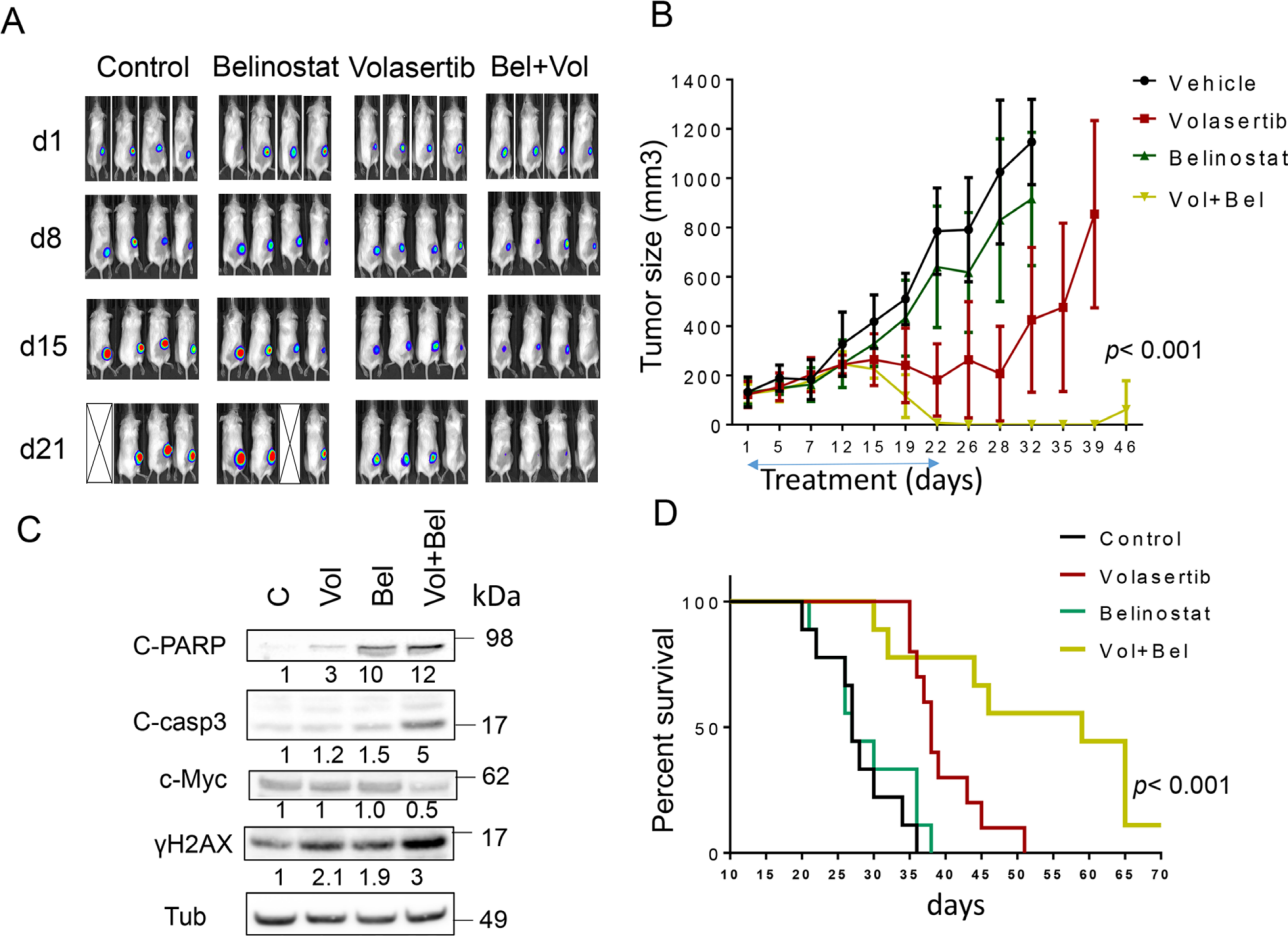
Co-treatment with volasertib and belinostat suppresses tumor growth in a murine xenograft model and prolongs animal survival. NOD/SCID-γ mice were subcutaneously inoculated in the right rear flank with 10 × 10^6^ U2932/Luc cells which stably express luciferase. Studies involved 9-10 mice per group. Treatment was initiated after the tumor were visualized, measured, and randomly grouped 10 days after injection of tumor cells. Belinostat was administrated at a dose of 80 mg/kg by i.p 5 days a week. Volasertib was administered at a dose of 12 mg/kg i.p once a week. Control animals were administered equal volumes of vehicle. **A.** Tumor growth was monitored twice weekly by injection of luciferin and imaged by the IVIS 200 imaging system. d=day, empty boxes represent deceased mice. **B.** Tumor size was measured by caliper twice weekly. Tumor volumes were calculated (length × width^2^/2) and plotted against days of treatment. The combination group exhibited significantly smaller tumor size than either single-agent volasertib or belinostat treatment (one-way Anova, p < 0.001). **C.** Mice were treated for 14 days, and after tumors reached 1 cm in diameter, a representative mouse in each group was sacrificed. Tumors were resected, homogenized and subjected to Western blot analysis for c-PARP, cleaved caspase-3, p-histone H3 and γH2A.X expression. **D.** Kaplan–Meier analysis was performed to analyze survival of animals. The survival of mice treated with the combination was significantly prolonged compared to mice treated with single agents (p< 0.001). Treatment was discontinued after day 21.

