# Supplementary material for: Synergistic interactions between PLK1 and HDAC inhibitors in non-Hodgkin's lymphoma cells occur in vitro and in vivo and proceed through multiple mechanisms
Source: Oncotarget. 2017 Feb 23;8(19):31478–93. doi: 10.18632/oncotarget.15649 (PMC5458223; doi:10.18632/oncotarget.15649)
Supplement: Supplementary file 1 [file oncotarget-08-31478-s001.pdf]

Synergistic interactions between PLK1 and HDAC inhibitors in non-Hodgkin's lymphoma cells occur *in vitro* and *in vivo* and proceed through multiple mechanisms

SUPPLEMENTARY FIGURES

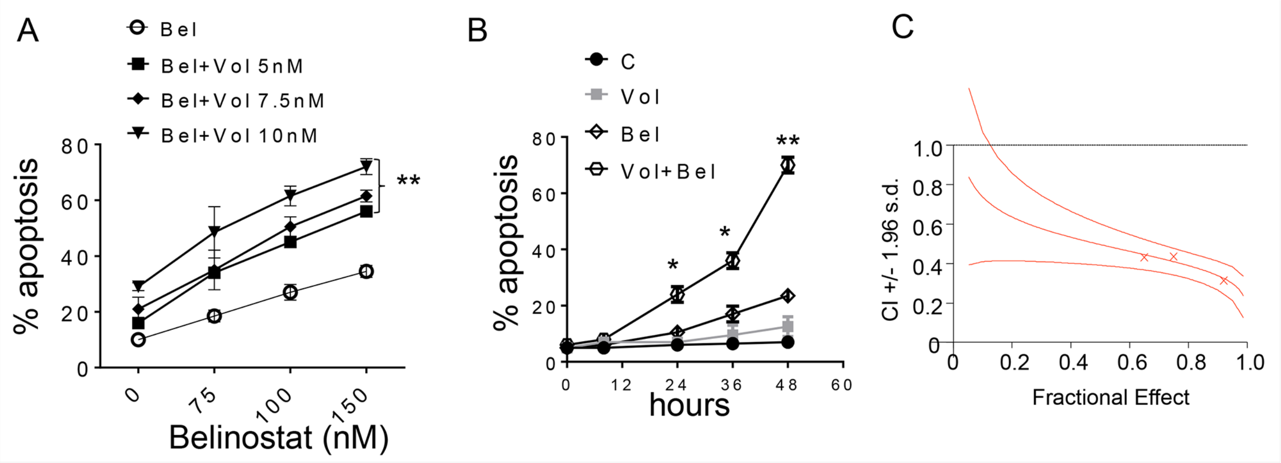

Supplementary Figure 1: Co-treatment with volasertib and belinostat synergistically induces cell death in double- hit lymphoma cells. A. OCI-Ly18 (double-hit) lymphoma cells were exposed to the indicated concentration of volasertib in the presence or absence of belinostat for 48 hours, after which cell death was assessed by 7-AAD uptake. **$p < 0.01$, significantly greater than values for single agent treatment. For these and subsequent studies, values represent the means ± S.D. for experiments performed in triplicate on at least 3 separate occasions. **B.** OCI-Ly18 cells were treated with volasertib (15 nM) or belinostat (250 nM) individually or in combination for the indicated intervals, after which the extent of cell death was determined. *$p < 0.05$, **$p < 0.01$, significantly greater than values for single agent treatment. **C.** OCI-Ly18 cells were treated with a range of volasertib and belinostat concentrations administered at a fixed ratio. At the end of 48 hr, the percentage of dead cells was determined by 7-AAD positivity. CI values were determined in relation to the fractional effect (FA) using Calcusyn software. CI values less than 1.0 correspond to a synergistic interaction.

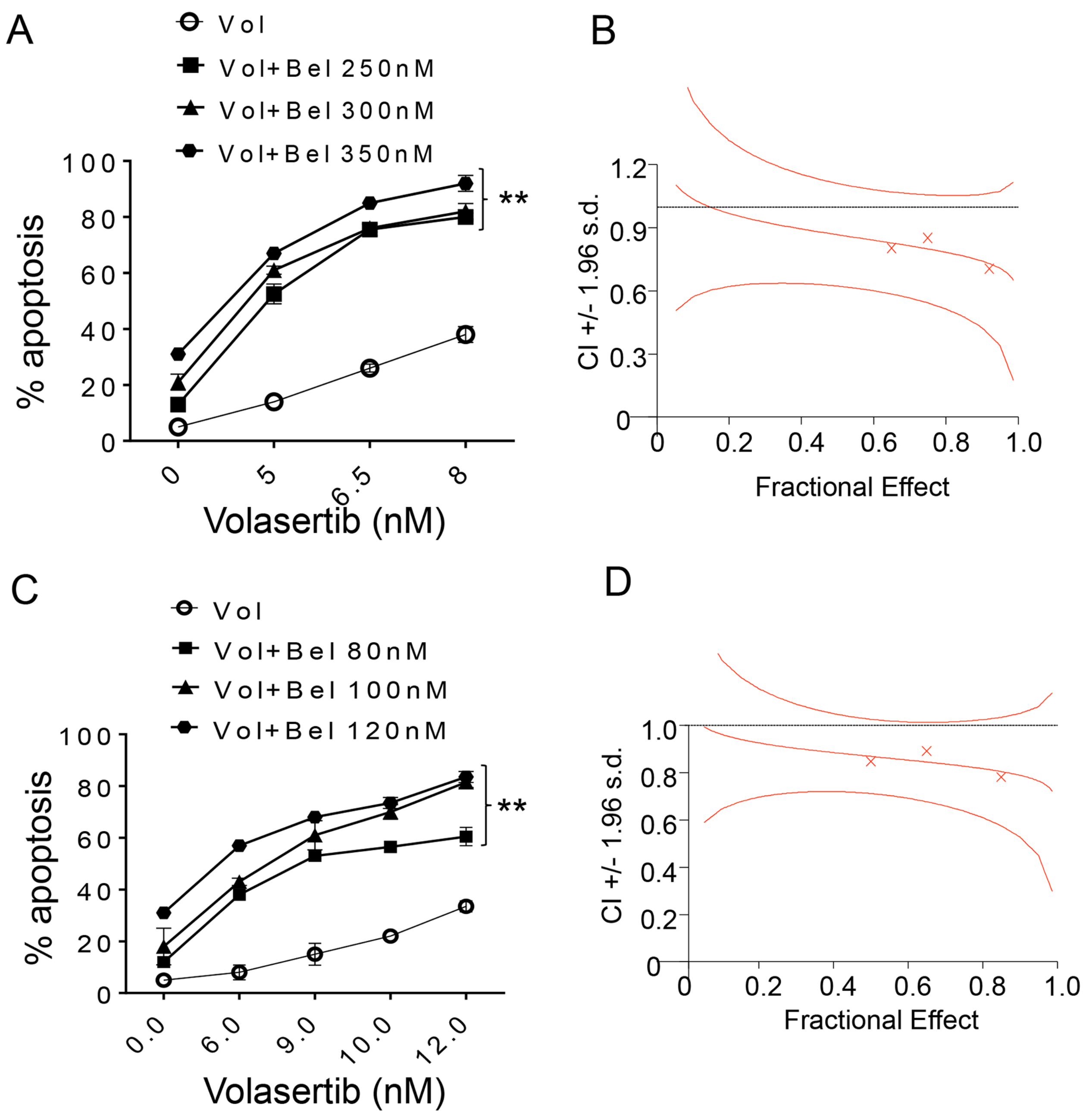

Supplementary Figure 2: Co-treatment with volasertib and belinostat synergistically induces cell death in the GC-subtype of DLBCL. A. SU-DHL8 cells (GC) were exposed to the indicated concentration of volasertib in the presence or absence of belinostat for 48 hours after which cell death was assessed by 7-AAD. **$p < 0.01$, significantly greater than values for single agent treatment. **B.** SU-DHL8 cells were treated with a range of volasertib and belinostat concentrations administered at a fixed ratio. After 48 hr, the percentage of apoptotic cells was determined by $7AAD^+$ staining. CI values were determined in relation to the fractional effect using Calcusyn software. CI values less than 1.0 correspond to a synergistic interaction. **C.** SU-DHL16 cells were exposed to the indicated concentration of volasertib in the presence or absence of belinostat for 48 hours, after which cell death was assessed by 7-AAD. **$p < 0.01$, significantly greater than values for single agent treatment. **D.** SU-DHL16 cells were treated with a range of volasertib and belinostat concentrations administered at a fixed ratio. After 48 hr, the percentage of apoptotic cells was determined by 7AAD positivity. CI values were determined in relation to the fractional effect using Calcusyn software. CI values less than 1.0 correspond to a synergistic interaction.

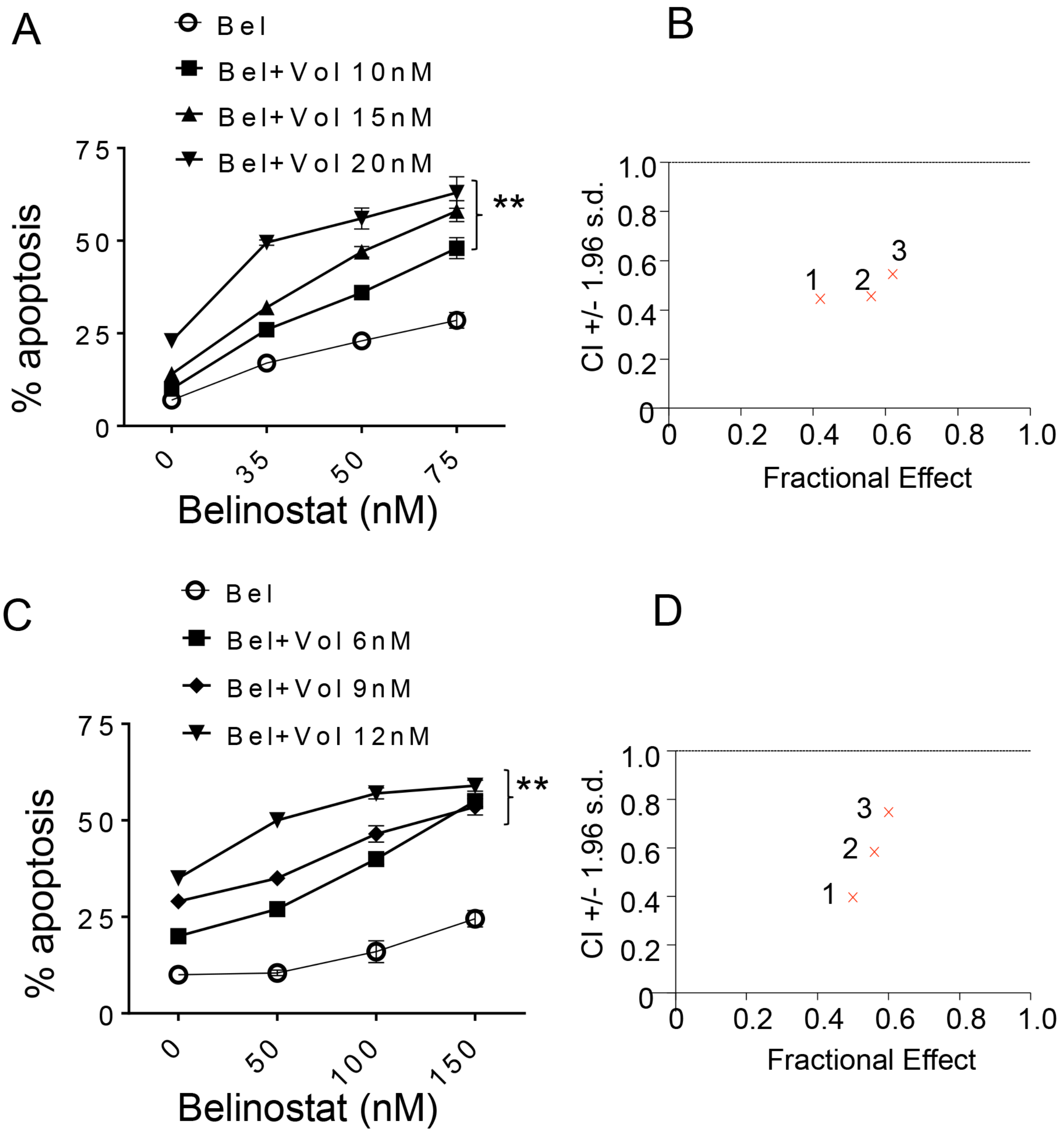

Supplementary Figure 3: Co-treatment with volasertib and belinostat synergistically induces cell death in ABC-DLBCL cells. A. HBL-1 cells (ABC-DLBCL) were exposed to the indicated concentration of volasertib in the presence or absence of belinostat for 48 hours, after which cell death was assessed by 7-AAD uptake. **$p < 0.01$, significantly greater than values for single agent treatment. **B.** HBL-1 cells were treated with a range of volasertib and belinostat concentrations administered at a fixed ratio. At the end of 48 hr, the percentage of dead cells was determined by 7-AAD positivity. CI values were determined in relation to the fractional effect using Calcusyn software. CI values less than 1.0 correspond to a synergistic interaction. **C.** U2932 cells (ABC-DLBCL) were exposed to the indicated concentration of volasertib in the presence or absence of belinostat for 48 hours, after which cell death was assessed by 7-AAD uptake. **$p < 0.01$, significantly greater than values for single agent treatment. **D.** U2932 cells were treated with a range of volasertib and belinostat concentrations. At the end of 48 hr, the percentage of dead cells was determined by monitoring 7-AAD positivity. CI values were determined in relation to the fractional effect using Calcusyn software. CI values less than 1.0 correspond to synergistic interactions.

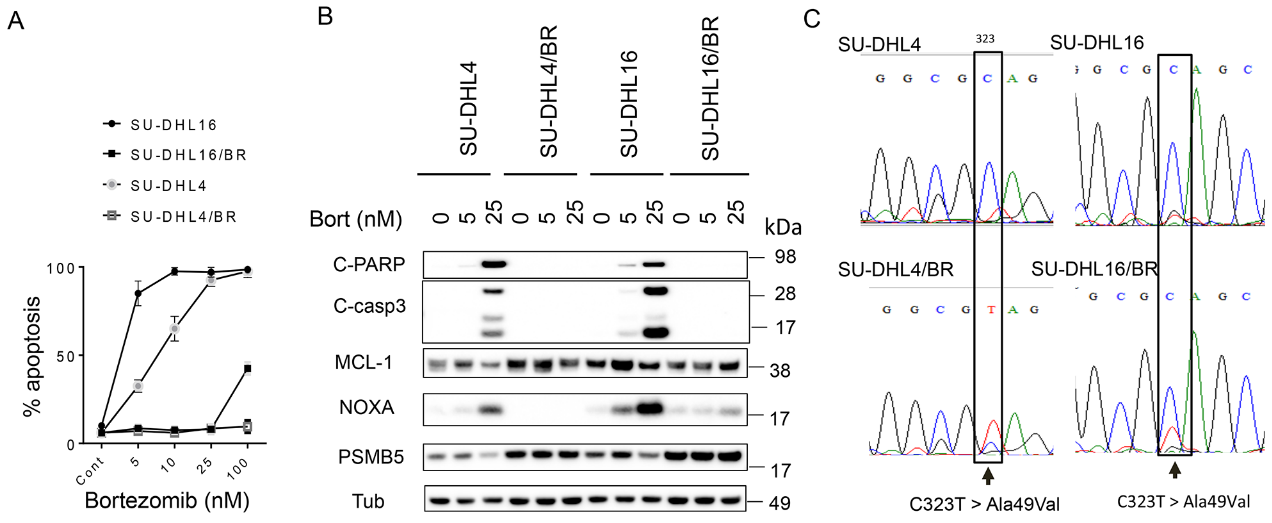

Supplementary Figure 4: Characterization of bortezomib-resistant cells and their response to volasertib and belinostat. **A.** SU-DHL16 and SU-DHL16/BR, SU-DHL4 and SU-DHL4/BR cells were exposed to the indicated concentration of bortezomib for 48 hours, after which the percentage of apoptotic cells was determined by 7-AAD positivity. **B.** Alternatively, cells were incubated with bortezomib (25 nM) for 4 hours after which cells were lysed and subjected to Western blot analysis to monitor c-PARP, cleaved caspase-3, PSMB5, Mcl-1 and NOXA expression. Each lane was loaded with 25 µg of protein; blots were subsequently re-probed and assayed for tubulin to ensure equivalent loading and transfer. **C.** Total RNA from SU-DHL16, SU-DHL4, SU-DHL16/BR and SU-DHL4/BR cells was extracted, followed by reverse transcription into cDNA. Exon II of the *PSMB5* gene was amplified and sequenced as described in Methods. A single point mutation was detected at nucleotide 323 varying from a C to T resulting in the Ala49Val mutation in SU-DHL4-BR and SU-DHL16-BR cells.

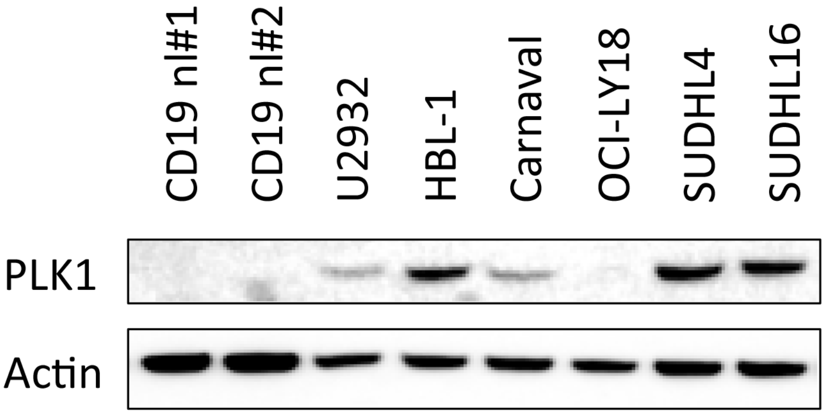

Supplementary Figure 5: Lymphoma cell lines exhibit high PLK1 expression compared to normal lymphocytes. Mononuclear from normal samples were isolated and enriched for the CD19^{+} population using an immunomagnetic bead separation technique, after which cells were lysed and proteins extracted. Expression of the indicated proteins was determined by Western blotting using the indicated antibodies. Each lane was loaded with 20 µg of protein; blots were stripped and re-probed with actin to ensure equivalent loading and transfer.

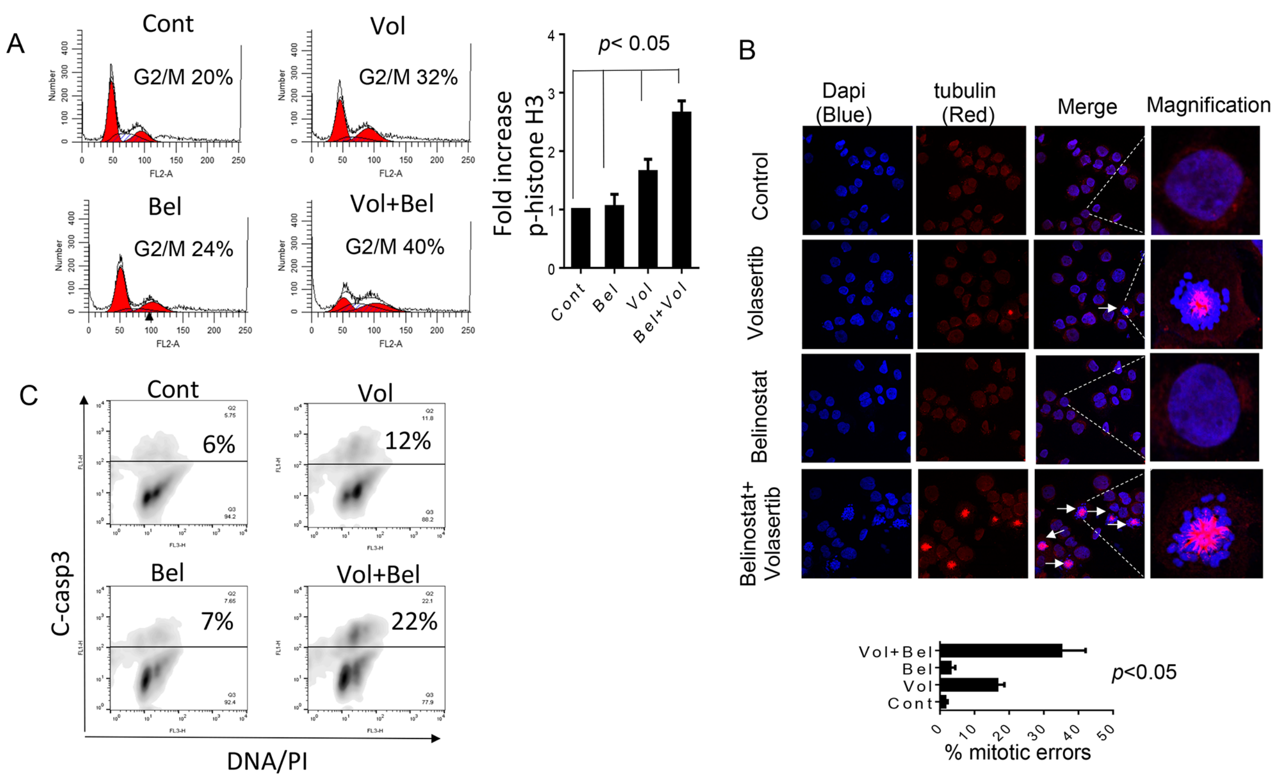

Supplementary Figure S6: The volasertib/belinostat regimen induces mitotic arrest, frequent mitotic errors and mitotic catastrophe in HBL-1 cells. A. HBL1 cells were exposed to 300 nM belinostat and 20 nM volasertib alone or in combination for 30 hr, after which cells were fixed and cell cycle distribution analyzed by flow cytometry (left panel). Cells treated as above were fixed and stained with p-Histone H3. The percentage of p-Histone H3-positive cells was then compared to values for single-agent treatment or untreated controls. (Values indicate fold increases compared to controls; right panel, $p < 0.05$). **B.** HBL1 cells were treated as above for 30 hr and immunofluorescence staining performed utilizing antibody directed against α-tubulin (red) and DNA counterstained with DAPI (blue). Arrows indicate cells displaying mitotic errors. High magnification images of a representative cell in each treatment group are shown in the right panels. A total of 100 cells per treatment were enumerated as normal or displaying mitotic errors including multi-polar spindles, improper tubulin alignment, or defective cytokinesis. The percentage of mitotic errors cells was determined and displayed in the lower panel with values for combined treatment significantly greater than those for control or single-agent treatment; $p < 0.05$. **C.** The distribution of apoptotic HBL1 cells in various cell-cycle phases following treatment with volasertib ± belinostat as above was then determined by combining staining for cleaved caspase-3 and DNA content (PI). Representative results are shown indicating caspase-3 activation in specific cell-cycle phases, including G_2M.

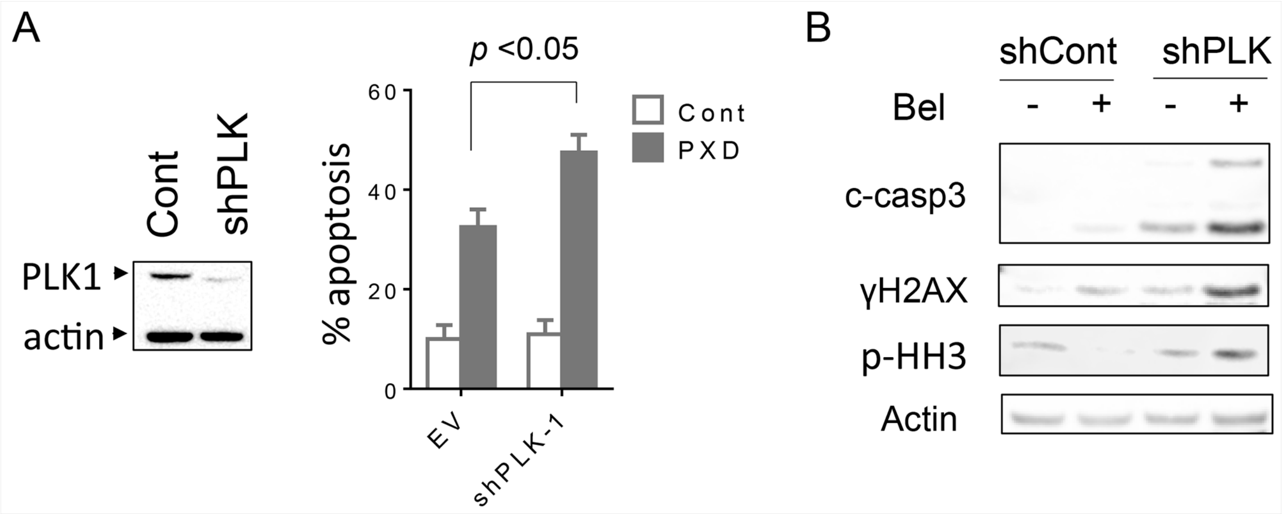

Supplementary Figure S7: Knockdown of PLK1 strikingly potentiates belinostat-mediated apoptosis in HBL-1 cells. **A.** HBL-1 cells were transiently transfected with shPLK1 or shCont vectors. PLK1 knockdown and control cells (left panel) were exposed to 400 nmol/L of belinostat for 48 hr, after which cell death was monitored by 7-AAD staining (right panel) ($p < 0.05$ for knock-down cells versus controls). **B.** shPLK1 and shCont cells were treated with 400 nmol/L of belinostat for 24 hr, after which Western blot analysis was performed to monitor cleaved caspase-3, p-Histone H3 and γH2A.X expression.

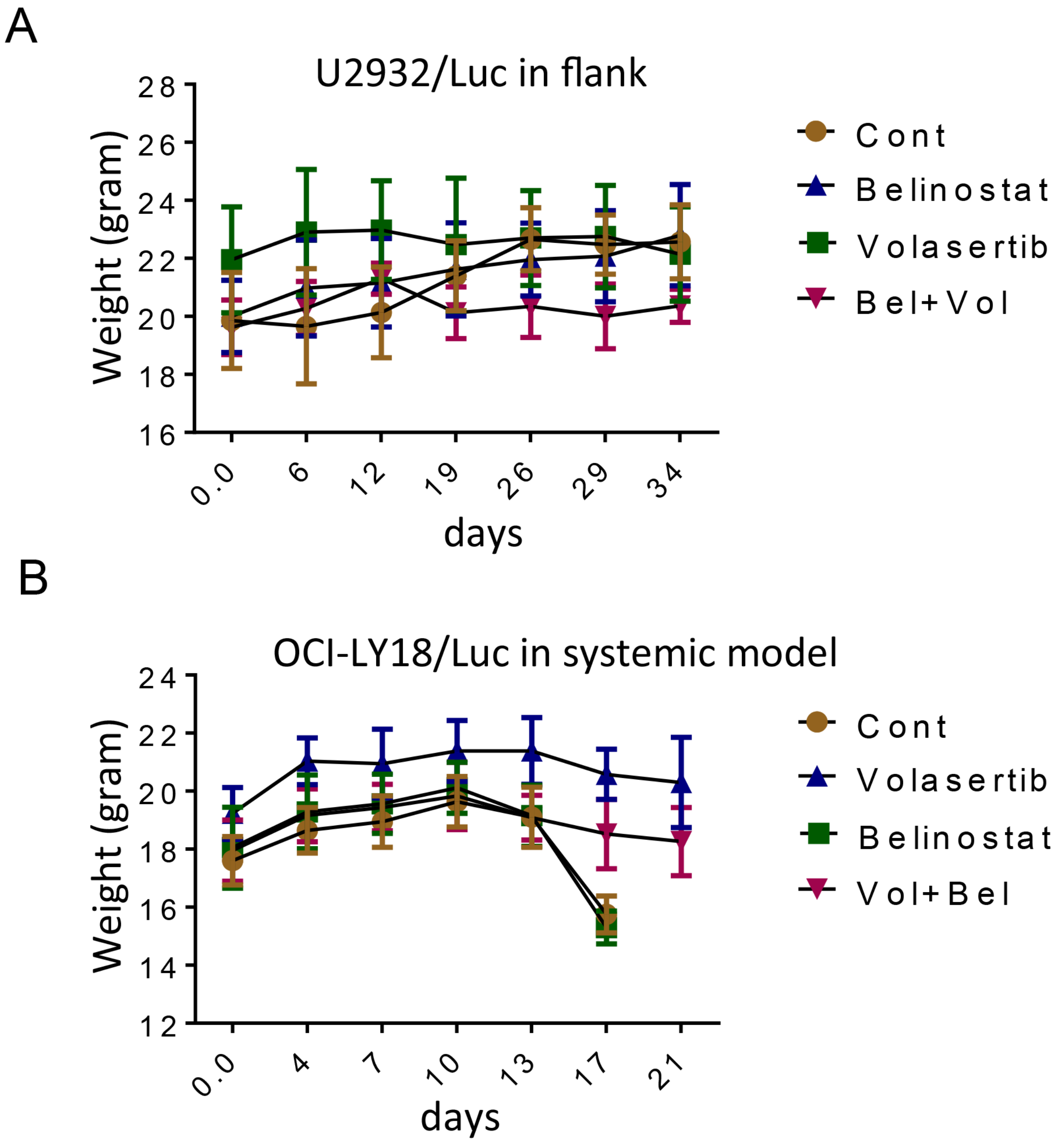

Supplementary Figure S8: Co-treatment with volasertib and belinostat does not cause significant weight loss in NSG mice. A. Weights of each mouse in the flank model experiment (Figure 6, U2932) were monitored twice a week and the mean weights for each group were plotted against days of treatment ($p > 0.05$ = no significant difference for the combination group values compared to single-agent treatment or control). **B.** Weights of each mouse in systemic model experiment (Figure 7, OCI-Ly18) were monitored twice a week and the mean weights for each group were plotted against days of treatment ($p > 0.05$, No significant differences were noted in the combination group values compared to single-agent treatment or control groups at day 14). Weight loss was only observed after day 13 in the control and belinostat group due to aggressive disease progression.
